# Integrative enrichment analysis: a new computational method to detect dysregulated pathways in heterogeneous samples

**DOI:** 10.1186/s12864-015-2188-7

**Published:** 2015-11-10

**Authors:** Xiangtian Yu, Tao Zeng, Guojun Li

**Affiliations:** School of Mathematics, Shandong University, Jinan, 250100 China; Key Laboratory of Systems Biology, Institute of Biochemistry and Cell Biology, Shanghai Institutes for Biological Sciences, Chinese Academy of Sciences, Cell Building Level 3, YueYang Road 320, Shanghai, 200031 China

## Abstract

**Background:**

Pathway enrichment analysis is a useful tool to study biology and biomedicine, due to its functional screening on well-defined biological procedures rather than separate molecules. The measurement of malfunctions of pathways with a phenotype change, e.g., from normal to diseased, is the key issue when applying enrichment analysis on a pathway. The differentially expressed genes (DEGs) are widely focused in conventional analysis, which is based on the great purity of samples. However, the disease samples are usually heterogeneous, so that, the genes with great differential expression variance (DEVGs) are becoming attractive and important to indicate the specific state of a biological system. In the context of differential expression variance, it is still a challenge to measure the enrichment or status of a pathway. To address this issue, we proposed Integrative Enrichment Analysis (IEA) based on a novel enrichment measurement.

**Results:**

The main competitive ability of IEA is to identify dysregulated pathways containing DEGs and DEVGs simultaneously, which are usually under-scored by other methods. Next, IEA provides two additional assistant approaches to investigate such dysregulated pathways. One is to infer the association among identified dysregulated pathways and expected target pathways by estimating pathway crosstalks. The other one is to recognize subtype-factors as dysregulated pathways associated to particular clinical indices according to the DEVGs’ relative expressions rather than conventional raw expressions. Based on a previously established evaluation scheme, we found that, in particular cohorts (i.e., a group of real gene expression datasets from human patients), a few target disease pathways can be significantly high-ranked by IEA, which is more effective than other state-of-the-art methods. Furthermore, we present a proof-of-concept study on Diabetes to indicate: IEA rather than conventional ORA or GSEA can capture the under-estimated dysregulated pathways full of DEVGs and DEGs; these newly identified pathways could be significantly linked to prior-known disease pathways by estimated crosstalks; and many candidate subtype-factors recognized by IEA also have significant relation with the risk of subtypes of genotype-phenotype associations.

**Conclusions:**

Totally, IEA supplies a new tool to carry on enrichment analysis in the complicate context of clinical application (i.e., heterogeneity of disease), as a necessary complementary and cooperative approach to conventional ones.

**Electronic supplementary material:**

The online version of this article (doi:10.1186/s12864-015-2188-7) contains supplementary material, which is available to authorized users.

## Background

Being a computational approach based on the prior knowledge, pathway enrichment analysis is widely used in the study of genotype-phenotype associations [1]. Biological pathway as a set of interactive genes (and a few of their interactions with biomolecules) produces particular cellular response/outcome by executing a series of functional cascades. It is curated by experts from wide range of science fields [2, 3] so that can supply more creditable functional details than general GO module or network module. Different from exploring the unknown or indeterminate functions by network module, pathway-centered analysis always makes an effort to capture the permutation of established functions (e.g., KEGG pathways [2, 3]) in the change of phenotypes (e.g., from normal to diseased). As a key approach of pathway-centered analysis, the pathway enrichment analysis or well-known gene set enrichment analysis (GSEA) [1] can identify dysregulated pathway by qualitatively measuring the changed status of a pathway [4].

In the pathway enrichment analysis, the dysregulation of a pathway is the most important issue [5], and should be mathematically defined and measured well [6]. It can estimate the conditional enrichment or status of a pathway, which is assumed to be associated with particular phenotypes. Current researches generally use genes with significantly differential expressions or differential correlations to evaluate the extent of the dysregulation of a pathway. One kind of conventional method is evaluating the dysfunction of pathways in different conditions [7–9], such as FiDePa (Finding Deregulated Paths Algorithm) [10], SPIA (Signaling Pathway Impact Analysis) [11] and iPEAP (Integrative Pathway Enrichment Analysis Platform) [12]. The other kind is using pathways to characterize individual samples [13, 14], like CORGs [15] and Pathifier [16]. Generally, all these methods focus on the genes with differential expression and their enrichments in pathways (i.e., the analysis in the context of differential expression) [17, 18], which assume the samples are of good purity in genotype-phenotype association study. However, in the study of complicated phenotypes, e.g., cancer study, a relevant problem is the samples with the same disease phenotype might be full of different unknown subtypes due to disease heterogeneity [19]. It is necessary to detect genes with new features observable in the complicated disease samples, and enhance the pathway enrichment analysis to be applicable in such previously unexpected situation [20].

**Fig. 1:**
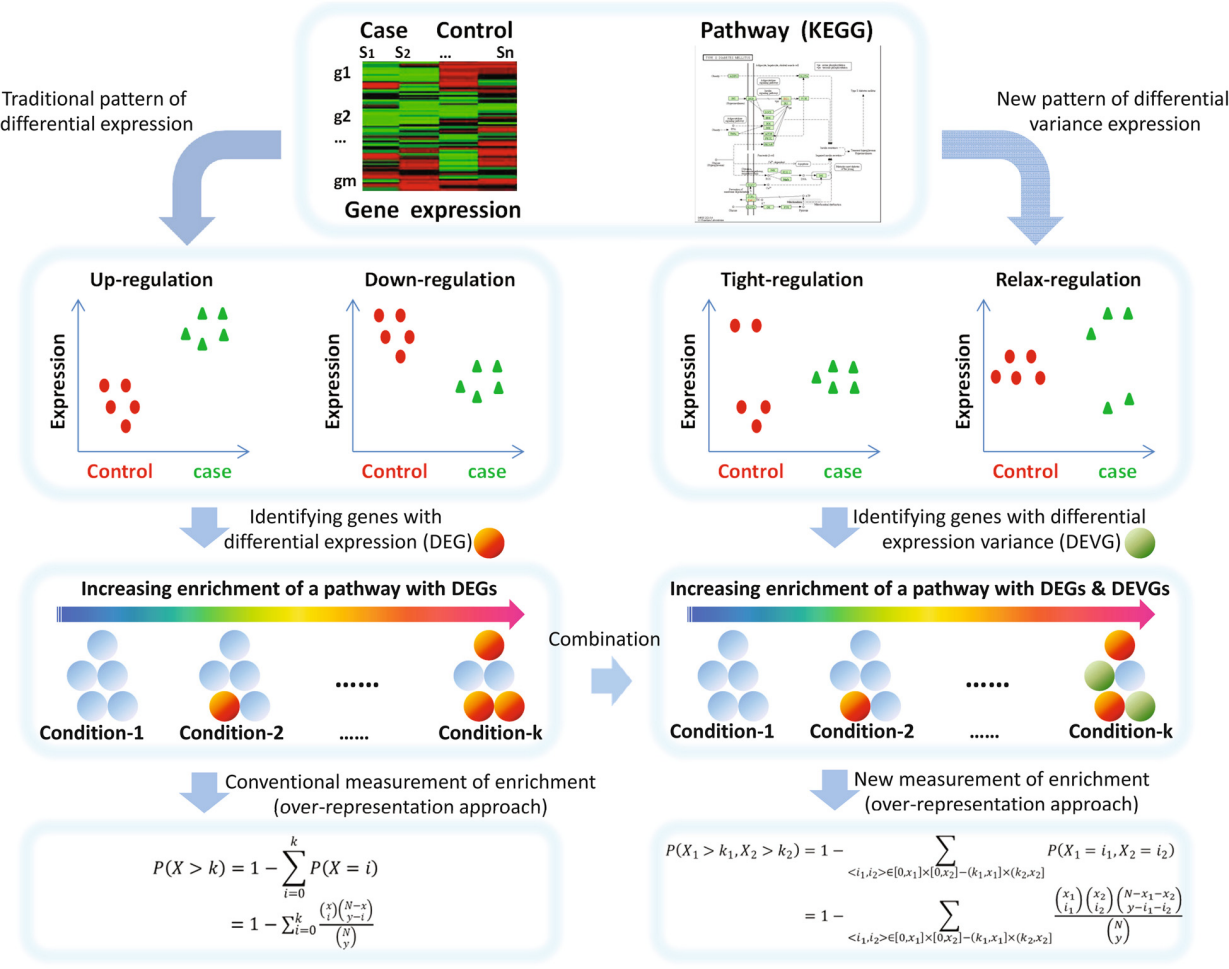
Major differences between the measurements of dysregulated pathways used in conventional enrichment analysis and integrative enrichment analysis (IEA)

Actually, there are new expression features extracted in recent studies, e.g., genes with differential expression variances [21, 22]. In the context of differential expression variance, it is still a challenge to measure the enrichment or status of a pathway. A solution to this problem can promote the efficiency of pathway enrichment analysis on genotype-phenotype association because it will consider more complete information about the expression changes of pathway genes. It can also provide new insights on the biological pathways by integrating additional expression and network features. In this work, we propose a multiple-label based enrichment analysis to detect such dysregulated pathways, which simultaneously takes into account the genes with differential expression (a label as DEGs) and genes with differential expression variance (the other label as DEVGs) together (Fig. 1).

Obviously, the hypothesis underlying IEA is that the dysregulated pathways involved in disease heterogeneity would be full of DEGs and/or DEVGs. That means the identified pathways by IEA would be disease pathways or their up-streams/down-streams (e.g., heterogeneity-relevant pathways or subtype-relevant pathways). However, current methods in pathway enrichment analysis only expect to give high-rank to disease pathways (e.g., target pathways in approach evaluation). When IEA identifies up-streams/down-streams of disease pathways, it further assistantly supplies a network of pathways to recover a global functional map and infer the associations among disease pathways and subtype-relevant pathways. Noted, the biological meaning of the edge in such network of pathways is the pathway crosstalk, which is just an important biological mechanism or functional relationship among pathways [23–26]. Conventional researches tend to simply determine a pathway crosstalk by the overlapped genes in two pathways [27], which disregard the statistical significance of the genes and interactions involved in the pathway crosstalk. By contrast, DEGs and DEVGs in one pathway can be used as seeds, and further detected their interactive genes in the candidate crosstalking pathways by a random walk restart algorithm [28]. The significance of a pathway crosstalk can be finally evaluated by the genes involved in this crosstalk as their enrichments in two pathways (i.e., the proposed multiple-label based enrichment).

Based on the above concepts and mathematical models, a new pathway-centered analysis framework, the integrative enrichment analysis (IEA), is implemented as (i) pathway enrichment score calculated by the hypergeometric test on differential genes (DEGs and DEVGs); (ii) pathway crosstalk ranked by the random walk and hypergeometric test on rewired molecule networks; (iii) pathway-phenotype association and subtype-factors determined by DEVGs in pathways. According to a previously established evaluation scheme [29], we found that, in particular cohorts (i.e., a group of real gene expression datasets from human patients), a few target disease pathways can be significantly high-ranked by IEA, which supplied the evidences of the deviation-based disease characteristics (i.e., disease subtypes), and IEA is more effective than other state-of-the-art methods in this condition. Furthermore, by a proof-of-concept study, we shows the details of IEA on analyzing real transcriptional data related to complex diseases, e.g., Diabetes and Colorectal cancer. IEA indeed captures the previously under-estimated pathways full of DEVGs and DEGs. These newly identified dysregulated pathways would be heterogeneity-relevant pathways and are found to be significantly linked to disease pathways (i.e., target pathways in conventional analysis) by estimated crosstalks. Many candidate subtype-factors are also recognized as DEVGs or pathways associated with the risk of subtypes of genotype-phenotype associations. Totally, IEA supplies a new way of over-representation approach [30] to carry on enrichment analysis in the complicate context of clinical application (i.e., differential expression and differential expression variance), and could be easily expanded to functional class scoring or pathway topology based approaches [31–34], which will be a necessary complementary and cooperative approach to conventional ones [35]. The Matlab scripts of the software named IEApackage and some alternative R scripts have been deposited in GitHub and accessed in https://github.com/bluesky2009/integrative-enrichment-analysis. This software has been developed and tested in Windows 7 or Windows 8, and Matlab 2010 or Matlab 2012.

## Methods

Generally, enrichment analysis includes three categories of methods: over-representation approach, functional class scoring and pathway topology based approaches. Although these methods are all focusing on evaluating the phenotype-associated pathway, they would be based on different hypothesis. This work and the proof-of-concept study are based on the over-representation approach, which measures the dysregulation extent of a pathway according to the number of dysregulated genes in this pathway. Traditional methods only evaluated the DEGs in a pathway; by contrast, IEA evaluates the DEGs and DEVGs in a pathway. Thus, the meaning of the statistic for the integration of IEA is as completely as possible to measure the dysregulation extent of a pathway according to the number of dysregulated genes (DEGs & DEVGs) in this pathway, which have been well defined and introduced in follows.

### Differential gene expression and differential expression variance

Given a gene *x* has expression profiles in control and case samples as X and X’ respectively, the expression variance of this gene in control and case condition are E((X-*u*)^2^) and E((X’-*u*’)^2^) respectively. Here, *u* and *u*’ are average expressions of gene *x* in control and case samples respectively. Then, the conventional criterion and measurement of genes with differential expression (named as DEGs) are:

1 $$ {\mathrm{H}}_0:\ \mathrm{E}\left(\mathrm{X}\right)=\mathrm{E}\left(\mathrm{X}'\right);\ {\mathrm{H}}_0\ \mathrm{rejected}; $$

where X or X’ are the original/raw expression levels. Noted, the differential expression includes *up-regulation* (the expressions of genes in case samples are larger than those in control samples) and *down-regulation* (the expressions of genes in case samples are less than those in control samples).

Except for these DEGs (e.g., genes rejected by Student’s *T*-test in significance test), the genes with differential expression variance are also discriminative features [21, 36]. The expression variance concerned features, e.g., bimodal gene expression, is already known as an important expression pattern in the control of a transition of biological systems [37], such as: disease development, cellular differentiation, and phase transition. However, the differential expression variance of genes has not been studied in a systematic way to the best of our knowledge, especially for its usage in the pathway enrichment analysis. The differential expression of genes, used in conventional enrichment analysis, requires the gene’s expressions under different conditions to distribute around different mean expression levels (seeing above formula 1). By contrast, differential expression variance of genes (named as DEVGs) can be defined as the genes’ deviations being significantly different under dissimilar conditions (deviation means the distances between a gene’s original expression levels and its mean expression level), such as:

2 $$ \begin{array}{l}{\mathrm{H}}_0:\ \mathrm{E}\left(\left|\mathrm{X}\hbox{-} u\right|\right)=\mathrm{E}\left(\left|\mathrm{X}'\hbox{-} u'\right|\right);\ {\mathrm{H}}_0\ \mathrm{rejected};\ \\ {}\mathrm{and}\ {\mathrm{H}}_0:\ \mathrm{E}\left(\mathrm{X}\right)=\mathrm{E}\left(\mathrm{X}'\right);{\mathrm{H}}_0\ \mathrm{not}\ \mathrm{rejected}\end{array} $$

where X or X’ is the original expression level, |X-*u*| or |X’-*u*’| is the relative expression level.

Noted, the differential expression variance includes *tight-regulation* (the expression variances of genes in case samples are less than those in control samples) and *relax-regulation* (the expression variances of genes in case samples are larger than those in control samples). And importantly, as defined above, the DEVGs have excluded DEGs, or there is no overlap between DEVGs and DEGs in this work. That means, when one gene has both differential expression and differential expression variance, this gene is thought as DEG in priority in order to be consistent with conventional analysis; and, of course, this kind of genes are worthy of deep research in future work.

Actually, given X or X’ satisfy normal distribution, |X-u| or |X’-u’| will be folded normal distribution, then the Wilcoxon rank sum test instead of Student’s *T*-test is used in the significance test of DEVGs.

### Integrative enrichment analysis in the context of differential expression variance

Obviously, the conventional enrichment analysis limits to estimate the extent of differential expression rather than differential expression variance. When considering the contribution of DEVGs on pathway’s dysregulation, it is necessary to refine the conventional approach to take into account the DEGs and DEVGs together. Naturally, an easiest strategy is to put DEGs and DEVGs together as the same dysregulated genes and use conventional hypergeometric test to obtain the *P*-value. However, this will disregard the respective distribution of DEGs and DEVGs in a target pathway and in the whole transcriptome. Thus, we extended the hypergeometric test on two kinds of enriched genes simultaneously as bellows. Our approach, noted as HT2 (hypergeometric test on the model of the drawn of two group balls), still depends on the hypergeometric distribution and uses *P*-value to measure the dysregulation of a pathway in the context of differential expression variance.

Briefly seen in Table 1, given there are expression data on total *N* genes, and *x*_*1*_ DEGs and *x*_*2*_ DEVGs selected respectively. For some pathway, *k*_*1*_ and *k*_*2*_ genes from pathway members (totally *y* genes) have differential expression and differential expression variance respectively. Then the significance of deregulated genes as DEGs or DEVGs enriched in this pathway can be estimated by formula 3. This *P*-value also ranges from zero to one. The less the *P*-value is, the larger dysregulation extent the pathway has, when the significantly larger number of genes in this pathway show differential expression or differential expression variance.

3 $$ \begin{array}{c}P\left({X}_1={k}_1,{X}_2={k}_2\right)=\frac{\left(\begin{array}{c}\hfill {x}_1\hfill \\ {}\hfill {k}_1\hfill \end{array}\right)\left(\begin{array}{c}\hfill {x}_2\hfill \\ {}\hfill {k}_2\hfill \end{array}\right)\left(\begin{array}{c}\hfill N-{x}_1-{x}_2\hfill \\ {}\hfill y-{k}_1-{k}_2\hfill \end{array}\right)}{\left(\begin{array}{c}\hfill N\hfill \\ {}\hfill y\hfill \end{array}\right)}\\ {}P\left({X}_1>{k}_1,{X}_2>{k}_2\right)=1-{\displaystyle \sum_{<{i}_1,{i}_2>\in \left[0,{x}_1\right]\times \left[0,{x}_2\right]-\left({k}_1,{x}_1\right]\times \left({k}_2,{x}_2\right]}P\left({X}_1={i}_1,{X}_2={i}_2\right)}\\ {} = 1-{\displaystyle {\sum}_{<{i}_1,{i}_2>\in \left[0,{x}_1\right]\times \left[0,{x}_2\right]-\left({k}_1,{x}_1\right]\times \left({k}_2,{x}_2\right]}\frac{\left(\begin{array}{c}\hfill {x}_1\hfill \\ {}\hfill {i}_1\hfill \end{array}\right)\left(\begin{array}{c}\hfill {x}_2\hfill \\ {}\hfill {i}_2\hfill \end{array}\right)\left(\begin{array}{c}\hfill N-{x}_1-{x}_2\hfill \\ {}\hfill y-{i}_1-{i}_2\hfill \end{array}\right)}{\left(\begin{array}{c}\hfill N\hfill \\ {}\hfill y\hfill \end{array}\right)}}\end{array} $$

**Table 1:** The statistic of DEGs and DEVGs for pathway enrichment analysis in the context of differential expression variance

	Pathway	Others	All
DEG	k_1_	x_1_-k_1_	x_1_
DEVG	k_2_	x_2_-k_2_	x_2_
Others	y-k_1_-k_2_	N + k_1_ + k_2_-x_1_-x_2_-y	N-x_1_-x_2_
All	y	N-y	N

### Estimating pathway crosstalks to link the dysregulated pathways identified by IEA and prior-known disease pathways

The first assistant down-stream analysis method of IEA is to link the dysregulated pathways identified by IEA and some prior-known disease pathways. Obviously, IEA tends to detect the dysregulated pathways related to disease subtypes. These pathways would be disease pathways as currently known, or the up-stream/down-stream of the disease pathways. Conventional pathway enrichment usually analyses single pathway rather than multiple ones. But, the pathway crosstalk, as a pair of pathways, also plays important roles in the change of phenotypes [25]. An enrichment analysis of such pathway crosstalk requires evaluating the enrichment of interactive genes from two pathways correspondingly. And the pathway map based on such estimated pathway crosstalks is just an additional computational method to assistantly supply a bridge between subtype-relevant pathways (i.e., IEA recognized pathways) and disease-relevant pathways (i.e., Target pathways from disease database KEGG).

Given several genes in a pathway as seeds, IEA uses random walk to find their partner genes in the other pathway. In fact, random walk with restart (RWR) is a well-known ranking algorithm for candidate gene prioritization [28]. It supplies the probability of searching the random walker at nodes in the steady state, so that, it can give a measure of proximity between source nodes (e.g., genes as seeds in a pathway) and other nodes in molecule network (e.g., genes in the candidate pathway with crosstalk).

Let *N* be the adjacency matrix of a gene network with node set *V* and edge set *E,* in which the element *N*_*ij*_ equals one if e(i, j) ∈ E (where e(i, j) represents the interaction between genes/nodes *i* and *j*), or zero otherwise. Based on the topological structure of the gene network, the transition matrix *T* can be calculated. Each element in the transition matrix is denoted as *T*_*ij*_ and represents the probability of transition from node *i* to node *j*. The value of *T*_*ij*_ can be given by one of two ways as follows, the first one is topology-weighted and the second one is correlation-weighted.

$$ {T}_{ij}=\left\{\begin{array}{c}\hfill \frac{N_{ij}}{d_i},\kern0.5em \mathrm{if}\ \mathrm{e}\left(\mathrm{i},\mathrm{j}\right)\in \mathrm{E}\hfill \\ {}\hfill 0,\kern0.45em  otherwise\ \hfill \end{array}\right., \kern0.5em \mathrm{where}\kern0.5em {d}_i={\displaystyle {\sum}_{j\in \mathrm{V}}{N}_{ij}} $$

$$ {T}_{ij}=\left\{\begin{array}{c}\hfill \frac{w_{ij}{N}_{ij}}{w_i},\kern0.5em \mathrm{if}\ \mathrm{e}\left(\mathrm{i},\mathrm{j}\right)\in \mathrm{E}\hfill \\ {}\hfill 0,\kern0.37em  otherwise\ \hfill \end{array}\right., \kern0.22em \mathrm{where}\kern0.5em {w}_i={\displaystyle {\sum}_{j\in \mathrm{V}}{w}_{ij}{N}_{ij}} $$

The RWR algorithm [28] updates the probability vectors by

$$ {P}_{k+1}=\left(1-\lambda \right)T{P}_k+\lambda {P}_0,\kern0.62em k>0 $$

where *T* is the transition matrix and *p*_*0*_ is the initial probability vector with the sum of the probabilities as one. In *p*_*0*_, all the source nodes are assigned equal probabilities and other nodes are given zero. *P*_∞_ is obtained when the algorithm is convergent. If *P*_∞_(*i*) > *P*_∞_(*j*), node *i* is thought to be more proximate to source nodes than node *j* does.

Thus, a two-way RWR approach (twRWR) is proposed to search the genes involved in two interactive pathways and estimate their enrichment for evaluating the pathway crosstalk. The steps of two-way RWR include:

(i) For each pathway *u*, its DEGs and DEVGs are used as source nodes/genes, and RWR is used to rank the genes in known molecule network, e.g., protein association network collected from STRING database [38].
(ii) In the high-ranked genes from above RWR analysis, the genes belonging to pathway *v* are the partner genes interactive with source genes. Based on the sources genes and their partner genes, the enrichment of those interactive genes (E_uv_) in pathways *u* and *v* can be evaluated by our HT2 approach, i.e., *P*-value in formula 3.
(iii) For every pathway, the analysis in steps (i) and (ii) is repeated. Then, given a pathway pair (*u*,*v*), it is a pathway crosstalk only when E_uv_ and E_vu_ are both significant. Finally, the map of pathways consist of those selected pathway crosstalks, where a node represents a pathway and an edge represents a pathway crosstalk.

### Screening subtype-factor of genotype-phenotype associations based on DEVGs and dysregulated pathways supplied by IEA

The second assistant down-stream analysis method of IEA is to screen subtype-factors according to the available clinical indices. As stated above, IEA focus on the DEVGs and their involved pathways, and these genes and pathways are thought as signatures of potential subtypes of heterogeneous samples. However, these hidden subtypes might have not been identified or formalized in clinics. To evaluate such new signatures or subtypes, one direct strategy is to measure the correlation between genetic signatures (e.g., DEVGs or dysregulated pathways) and clinical indices (e.g., age or bmi). If one signature is significantly related to some clinical index, the subtype represented by such signature would be medical meaningful as to be observable in clinics and this signature is also called as subtype-factor related to particular clinical index. The approach to identify such subtype-factors is described in bellows.

For each pathway, its DEVGs are used to group case (or control) samples into two clusters, when the case (or control) samples have high varying expression compared to control (or case) samples. That means these genes have over-expression in one group of samples and under-expression in the other group of samples. This pathway would be a candidate subtype-factor when these two sample clusters are discriminative on some clinical index. On this condition, a clinical subtype of samples is thought to be related to a given clinical index, which is represented by a subtype-factor (e.g., a DEVG or a dysregulated pathway from IEA). Obviously, the clinical subtype of a particular sample might be contributed by many subtype-factors (i.e., many pathways). Given a known phenotype (e.g., a clinical index), a few subtype-factors correlated with this phenotype can be found, although which just reveals only the tip of the iceberg for the subtypes of genotype-phenotype associations.

Particularly, different from conventional un-supervised clustering for subtype identification, a supervised-like clustering approach (SLC) is proposed to identify subtype-factors on the level of pathways. Firstly, the case samples can be grouped into two clusters according to their features’ values (i.e., DEVGs’ expressions) compared to those values of control samples: on each feature (DEVG), one group of samples have larger values than controls meanwhile the other group of samples have less values than the same controls, or vice versa. That means, a hyperplane determined by a few control samples could separate the samples space into two sub-spaces, and case samples in each of two sub-spaces are grouped into one cluster. Secondly, some clinical information of samples can be used to evaluate the potential subtype represented by such two clusters of case samples. If the clinical values of these two groups of samples have significant difference, a clinical subtype of genotype-phenotype association (e.g., the correlation between clinical indices and pathway DEVGs) is identified and the corresponding pathway is a subtype-factor corresponding to the given clinical index.

Practically, the SLC algorithm on a pathway is implemented as bellows:

(i) Discrete the expressions of DEVGs of case samples into binary vector based on the values of controls: for a DEVG, if its expression value is larger than the mean of controls, it is one in the binary vector; otherwise, it is zero.
(ii) Clustering case samples based on the binary vectors by conventional methods as hierarchical clustering or K-means, which obtains two sample clusters.
(iii) Calculating the significance of difference between clinical indices among above two sample clusters. If the difference is significant, this pathway is identified as a subtype-factor of the association between the given pathway and clinical index.

## Results and discussion

### The evaluation of biological meaning of IEA by method comparison

IEA is proposed to evaluate dysregulated pathways by differential gene expression and differential expression variance together. Differential expression variance has been reported as a new and important expression change during a phenotype change [36], e.g., diseases. In this work, the biological hypotheses underlying IEA is that, the dysregulated pathways full of genes with differential expression variance would be subtype-relevant pathways. Although subtype-relevant pathways for particular complex disease are unclear in current pathway databases, e.g., KEGG, it is still able to investigate if prior-known disease pathways in KEGG would be subtype-relevant and if IEA can identify them. In the previous study of gene-set analysis [29], a comparison scheme has been built to evaluate the performances of different enrichment analysis methods (e.g., ORA or GSEA) based on multiple expression datasets about complex diseases. Different from previous general comparison, we focus on the comparisons by approach-specific datasets, in order to mainly evaluate the biological meaning of IEA.

According to the comparison protocol [29], we ran total eight representative enrichment analysis methods on 36 GEO datasets with target pathways in KEGG, and obtained the rank of target pathway estimated by each method on each dataset; then, for each dataset, we rank the eight methods according to their prioritization performance or sensitivity performance [29], and this dataset is assigned as a specific-data for the Top-K methods (K is set 3); thus, all specific-data for one method can consist of K-order approach-specific dataset. Generally, on one method’s approach-specific dataset, this method should have best or comparable performances than other methods, so that, the biological characteristics assumed by this given method would significantly displayed on these datasets. Therefore, we can use this strategy to investigate the biological meaning of IEA in real datasets. In bellows, we firstly summarize the biological hypothesis hold by different state-of-the-art enrichment analysis methods and their respective quantitative measurements, and then discuss the comparison between IEA and others.

(i) PLAGE: it assumes the activity of pathway rather than the expression of pathway genes determines the activated or inhibited status of pathways under different conditions; and the pathway activity is measured by an activity score as the weights of a metagene extracted from all pathway genes by SVD (singular value decomposition) [39].
(ii) GSVA: it proposes the change of pathway activity between control and case should be evaluated at the level of samples, e.g., considering the variation of pathway activity over a sample population; and the pathway activity is measured by so-called GSVA score as a function of the expressions of genes inside and outside the pathway, and these scores are assessed similarly as GSEA by using the Kolmogorov-Smirnov (KS) like random walk statistic [40].
(iii) PADOG: it assumes that, if the genes highly specific to a given pathway occur differential expressions, the respective pathway would be truly relevant in that condition; thus, a new gene set score is calculated as the mean of absolute values of weighted moderated gene t-scores where the gene weights are designed to be large for the genes appearing in few pathways and small for genes that appear in many pathways [41].
(iv) GLOBALTEST: it holds an assumption that, if a group of genes (e.g., pathway genes) can be used to predict the clinical outcome, the expression patterns of such gene group must differ for dissimilar clinical outcomes; thus, it uses generalized linear model to give one *P*-value for a group of genes, not a *P*-value for each gene, which can be applied to estimate the enrichment of a given pathway [42].
(v) MRGSE: it proposes that the high ranks of expression changes (e.g., fold-change) of genes can indicate the differential expression of a set of genes (e.g., pathway genes); and the enrichment score or the test statistic of a pathway is the mean rank of this gene set, i.e., the average of the ranks of t-statistics of pathway genes [43].
(vi) GSA: it is similar to GSEA, and proposes two improvements as the maximal average statistic for summarizing gene-sets, and restandardization for accurate enrichment inferences [44].
(vii) ORA: it takes into account the number of differentially expressed genes observed in a pathway as indicators of pathway states; generally, it uses a basic contingency table to test the association between the differential expression status of a gene (e.g., differentially expressed gene, or not) and its membership in a given gene set (e.g., pathway gene, or not), which can be measured by the *P*-value of a hypergeometric test [45].
(viii) IEA: it is proposed in this work to generally consider the contribution of expression variance in a dysregulated pathway; as one implementation, this work takes into account the number of DEGs and DEVGs observed in a pathway as indicators of pathway states; it is designed to test the association between the differential expression/differential expression variance status of a gene and their memberships in a given gene set, which can be measured by the *P*-value from proposed HT2 approach in this work.

**Fig. 2:**
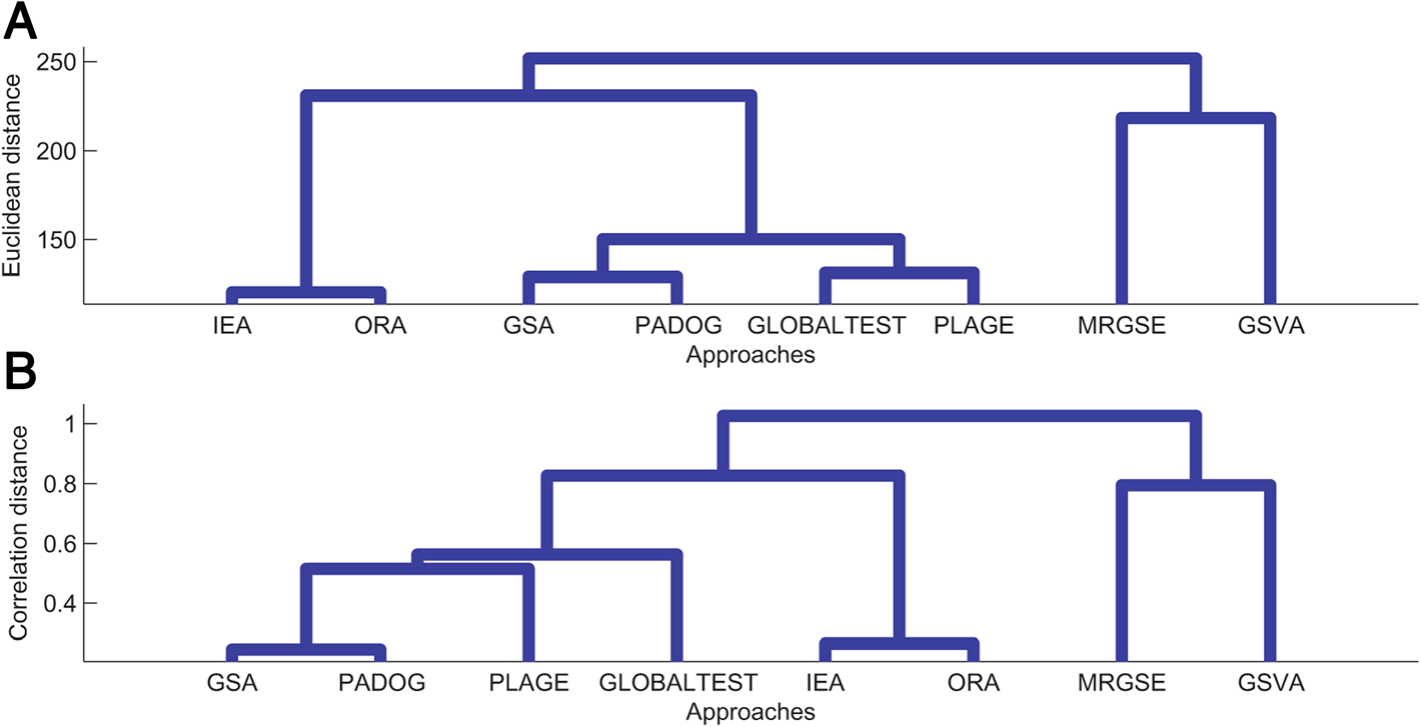
Category of representative gene set analysis approaches based on clustering of prioritization performance. **a** Method clustering based on Euclidean distance of ranks of all pathways. **b** Method clustering based on Correlation distance of ranks of all pathways

**Fig. 3:**
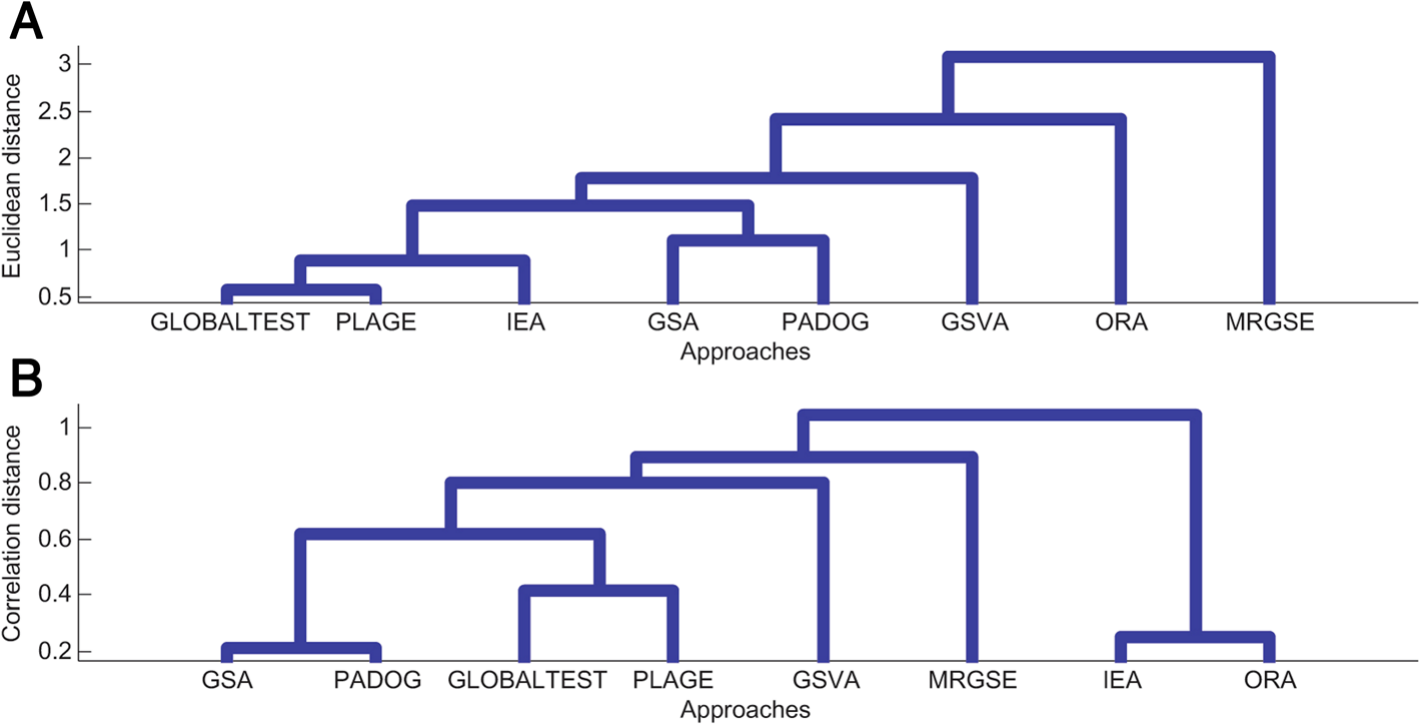
Category of representative gene set analysis approaches based on clustering of sensitivity performance. **a** Method clustering based on Euclidean distance of *P*-values of all pathways. **b** Method clustering based on Correlation distance of *P*-values of all pathways

First of all, we can cluster the above eight approaches by their performances on all datasets to investigate the general association among different methods. As shown in Figs. 2 and 3, the similarity among any two methods is measured by four kinds of criterion: the first one is whether the ranks given by two methods on the same dataset are also the same (i.e., Euclidean distance on ranks in Fig. 2a); the second one is whether the ranks given by two methods have the same change tendency among different datasets (i.e., Correlation distance on ranks in Fig. 2b); the third one is whether the *P*-values given by two methods on the same dataset are also the same (i.e., Euclidean distance on *P*-values in Fig. 3a); and the last one is whether the *P*-values given by two methods have the same change tendency among different datasets (i.e., Correlation distance on *P*-values in Fig. 3b). Obviously, GSA and PADOG are both based on conventional GSEA, so that they are similar; the proposed IEA is based on ORA, thus, they also have similar performances on different datasets; PLAGE and GLOBALTEST are closely clustered together, one reason is that they both estimate a score from all pathway genes rather than individual genes (i.e., PLAGE uses weights of a metagene extracted from all pathway genes by SVD, and GLOBALTEST uses generalized linear model to give one *P*-value for a group of genes); in addition, MRGSE and GSVA are much different, and also different form other methods, which is possibly because they have specific design principles on the measurement of pathway dysfunctions, i.e., MRGSE combines the t-statistics of individual pathway genes meanwhile GSVA uses a score as a function of the expressions of genes inside and outside a pathway.

Then, we directly grouped the datasets according to the performance of a given method, e.g., some datasets are included as K-order IEA-specific datasets, only when the rank of IEA performance compared to all methods are in the Top-K on these datasets, where K is set 3 in this study. To quantify the performance, sensitivity (i.e., *P*-value) and prioritization (i.e., rank) are adopted as previously [29]. In previous evaluation on these datasets, PADOG displays consistently comparable performance with other methods, meanwhile, PLAGE, GLOBALTEST and MRGSE have the best performances on some categorise of datasets [29], which already suggest the existence of approach preferences. Thus, the categories of datasets induced in this work can indicate potential preferences of different methods on particular datasets. Of course, on the preference-specific datasets (i.e., approach-specific datasets), the preferred method should have the best performance; meanwhile, some other methods would have comparable performances. Therefore, this new evaluation scheme can supply evidences for two hypotheses: one is that the expression features of a pathway measured by a given method actually have biological meaning, and are existing or observable in real datasets corresponding to particular phenotypes (e.g., complex diseases); the other one is that a method has significant preferences (i.e., highest performance) on a group of datasets (i.e., diseases), which is comparable or not by other methods.

As seen in Table 2 for prioritization performance, every method shows its preferences on particular datasets (The list of these 3-order approach-specific datasets is in Additional file 1: Table S1); in addition, the method comparison on more strict specificity as 2-order approach-specific datasets (K = 2) and more weak specificity as 4-order approach-specific datasets (K = 4) have also been done and reported in Additional file 2: Table S2 and Additional file 3: Table S3. Combined these results together, it is easily to see that: IEA, ORA and PADOG have significant preferences, due to their highest performances than all other methods on their correspondingly preferred datasets; GLOBALTEST, GSVA, PLAGE and MRGSE also have significant preferences on datasets, although sometimes PADOG could have comparable performances with them (e.g., PADOG and MRGSE are comparable when K = 3 in Table 2, but not when K = 2 in Additional file 2: Table S2; or PADOG are comparable to GLOBALTEST, GSVA, PLAGE when K = 4 in Additional file 3: Table S3, but not when K < 4); besides, GSA is challenged by PADOG, because on the GSA-specific datasets, PADOG always achieves similar or better performances, but not vice versa.

**Table 2:** The prioritization performance about method comparison on approach-specific datasets (K = 3)

ID	GSA-specific	PADOG-specific	IEA-specific	MRGSE-specific	ORA-specific	GLOBALTEST-specific	GSVA-specific	PLAGE-specific
**GSA**	**(14.93,12.82)**	(25.57,25.66)	(44.47,29.40)	(37.43,26.53)	(45.72,29.72)	(32.23,31.44)	(22.52,26.76)	(33.26,22.45)
**PADOG**	***(11.98,12.48)***	**(10.06,9.34)**	(29.23,23.83)	***(19.32,15.79)***	(26.56,20.69)	(16.84,21.56)	(15.47,12.87)	(18.80,21.06)
**IEA**	(52.05,28.86)	(51.15,26.26)	**(18.55,11.72)**	(68.01,16.79)	(34.14,15.48)	(41.20,26.26)	(53.04,29.63)	(51.87,31.13)
**MRGSE**	(51.97,30.59)	(51.23,29.03)	(65.81,31.64)	**(24.67,16.48)**	(74.35,19.55)	(50.63,28.47)	(45.72,27.77)	(49.39,26.17)
**ORA**	(47.22,31.35)	(48.91,28.29)	(24.80,15.05)	(69.98,17.56)	**(23.56,14.62)**	(52.38,23.61)	(38.76,28.78)	(53.60,26.98)
**GLOBALTEST**	(36.52,21.70)	(31.10,18.93)	(30.39,15.78)	(35.44,18.29)	(41.53,18.15)	**(14.66,16.19)**	(45.29,21.23)	(26.18,22.77)
**GSVA**	(33.56,24.84)	(47.83,28.90)	(59.09,26.48)	(52.99,27.27)	(52.52,25.58)	(61.99,29.17)	**(13.42,10.25)**	(62.49,25.01)
**PLAGE**	(26.44,16.96)	(29.07,20.48)	(42.06,34.24)	(35.48,19.88)	(45.77,24.91)	(30.15,27.15)	(31.86,16.67)	**(13.61,11.04)**

The performance of an approach on its specific dataset is highlighted in bold. And the performance of comparable approaches on some specific dataset is highlighted in bolditalic

And seen in Table 3 and Additional file 4: Table S4 for sensitivity performance, again, every method shows its preference on particular datasets (The list of these 3-order approach-specific datasets is in Additional file 5: Table S5). It seems that GLOBALTEST and PLAGE have generally comparable performances with other methods according to their performances on many preferred datasets of other methods. Even though, IEA shows the best performance on the IEA-specific datasets, and is better than ORA on the ORA-specific datasets. This fact strongly suggests that, IEA actually can detect dysregulated pathways, and displays competitive performance than conventional methods in current evaluation scheme when those dysregulated pathways are just target pathways (e.g., disease pathways in KEGG); besides, IEA is realized based on the conventional ORA, and improves ORA on the sensitivity performance, which would just be contributed by considering the new feature genes as DEVGs in dysregulated pathways.

**Table 3:** The sensitivity performance about method comparison on approach-specific datasets (K = 3)

ID	GSA-specific	PADOG-specific	IEA-specific	MRGSE-specific	ORA-specific	GLOBALTEST-specific	GSVA-specific	PLAGE-specific
**GSA**	**(0.10,0.12)**	(0.22,0.28)	(0.35,0.29)	(0.042,0.014)	(0.48,0.40)	(0.25,0.27)	(0.19,0.22)	(0.21,0.22)
**PADOG**	(0.11,0.15)	**(0.051,0.11)**	(0.24,0.20)	(0.057,0.011)	(0.41,0.28)	(0.14,0.17)	(0.13,0.12)	(0.12,0.15)
**IEA**	(0.11,0.17)	(0.13,0.13)	**(0.044,0.057)**	(0.12,0.14)	***(0.012,0.0043)***	(0.092,0.11)	(0.12,0.13)	(0.10,0.12)
**MRGSE**	(0.73,0.19)	(0.46,0.31)	(0.59,0.27)	**(0.020,0.012)**	(0.56,0.077)	(0.47,0.32)	(0.46,0.31)	(0.50,0.32)
**ORA**	(0.24,0.23)	(0.49,0.27)	(0.24,0.21)	(0.50,0.60)	**(0.13,0.085)**	(0.39,0.29)	(0.37,0.28)	(0.40,0.29)
**GLOBALTEST**	(0.11,0.13)	***(0.013,0.028)***	(0.052,0.084)	***(0.00011,0.00015)***	***(0.083,0.10)***	**(0.011,0.044)**	(0.037,0.086)	(0.025,0.073)
**GSVA**	(0.11,0.11)	(0.32,0.28)	(0.33,0.26)	(0.060,0.080)	(0.21,0.23)	(0.24,0.26)	**(0.013,0.017)**	(0.21,0.25)
**PLAGE**	***(0.097,0.11)***	(0.063,0.18)	(0.095,0.15994)	***(0.010,0.014)***	(0.19,0.10)	(0.036,0.11)	(0.034,0.076)	**(0.022,0.066)**

The performance of an approach on its specific dataset is highlighted in bold. And the performance of comparable approaches on some specific dataset is highlighted in bolditalic

Finally, above dataset-driven method comparison supplies new insights on the performance specificities of many gene-set approaches, especially for IEA; and also supports the importance and biological meaning of dysregulated pathways identified by IEA. We can draw following conclusions:

(i) Although a few methods have consistent performances on many datasets (e.g., GLOBALTEST and PLAGE on sensitivity performance, or PADOG on prioritization performance, as shown in both this study and previous work [29]), different method still have their preferences on the expression characteristics of dysregulated pathways, so that each method can achieve significantly better performances on their specific datasets rather than all datasets. Especially, on the IEA-specific datasets, IEA indeed are the best one than other methods; and even on the ORA-specific datasets, IEA is better than ORA on sensitivity performance. On the IEA-specific datasets, the target pathways or disease pathways are possibly just the subtype-relevant pathways, so that, IEA have competitive performance in the comparison scheme. Therefore, the expression variance focused by IEA is actually full of biological meaning, and will help IEA to detect new dysregulated pathways, e.g., subtype-relevant pathways. In addition, the complex diseases concerned in IEA-specific datasets actually already have reports about the existence of subtypes on genetic level, such as dilated cardiomyopathy [46], renal cancer [47], prostate cancer [48], colorectal cancer [49], and thyroid cancer [50].
(ii) Every method, or every method category, can actually capture particular dysregulated pathways. When the target pathway of a dataset just displays the expression characteristics focused by an approach, such approach would have better performance on this dataset. On some specific datasets preferred by other methods, IEA should face two conditions: one is under the condition that the target pathway is not subtype-relevant pathway, and IEA will be underestimated but has supplied a useful down-stream analysis (i.e., map of pathways) to assistantly link the target pathway and potential subtype-relevant pathway identified; the other one is under the condition that the target pathway is a subtype-relevant pathway, and IEA should be further enhanced by integrating expression variance with other pathway pattern (e.g., linear model in GLOBALTEST or weights of overlapping genes in PADOG), which is worthy of study in future.

### A proof-of-concept study of IEA on transcriptional analysis of complex diseases (diabetes)

IEA has been applied to detect the biological malfunction of complex diseases (e.g., Type II Diabetes) on the pathway level rather than gene level. IEA, as a pathway-centred analysis approach, not only supplies the conventional pathway enrichment analysis but also extracts divergent pathway associations, such as: pathway & disease genes, pathway & pathway (i.e., pathway crosstalk), and subtypes of pathway & clinic (i.e., genotype-phenotype association).

Firstly, it is the data pre-procession. Data needed in IEA have been prepared from the public resources: The gene expression data of human islets from non-diabetic and diabetic were downloaded through GEO [51]; there are two datasets, the main dataset GSE41762 [52] contains samples from 57 non-diabetic and 20 diabetic with 20950 genes, and the replicate dataset GSE38642 [53] contains samples from 54 non-diabetic and 9 diabetic with 19514 genes; the gene lists of 186 KEGG pathways are obtained from GSEA package [1]; the human protein interaction network (PIN) are extracted from STRING database [38] with confidence score no less than 0.9; four clinical indices are also obtained from the supplementary of original study [52] as sex, age, bmi, and HbA_1c_; meanwhile, diabetes associated genes are searched from GeneCards database [54].

Secondly, it is the main step of IEA. Different scores of pathway enrichment are calculated: (i) the conventional score (ORA) as *P*-values of hypergeometric distribution of DEGs in a pathway; (ii) the conventional score (GSEA) as *P*-values of estimated pathway enrichment; (iii) the new score (IEA) as *P*-values of hypergeometric distribution of differential genes (integrating DEGs and DEVGs) in a pathway calculated by the proposed HT2 approach. For ORA or IEA, the thresholds of *P*-value of significance test on DEGs or DEVGs are both set as 0.05, and adopted a pervious strategy to select those feature genes [29]: 1) select all genes with FDR adjusted *p*-values no more than 0.1; 2) if the genes selected are less than 200, re-select all genes with *P*-values no more than 0.05 and fold-change no less than 1.5; 3) if the genes selected are still less than 200, directly use the top 1 % of genes ranked by *P*-values from least to largest.

Thirdly, it is one assistant step of IEA. Pathway crosstalks are evaluated by two-way RWR approach. The interactions selected from PIN consist of differential network [55], where the selected interactions have significant correlation difference between diabetic and non-diabetic groups. On this differential network, two-way RWR approach is used to find the pathway crosstalks. The most significant pathway crosstalks (the threshold of *P*-value of significance test is set as 0.001 strictly) consist of the map of pathways. Besides, the enrichments of pathway genes or disease-associated genes in the high-ranked genes of RWR are also analyzed and evaluated by AUC [56], which support the efficiency of RWR on pathway-related analysis.

Fourthly, it is the other assistant step of IEA. The DEVGs in each pathway are used to group samples in two clusters by SLC approach. For each clinical index from sex, age, bmi, and HbA_1c_, its subtype-factors are identified. As comparisons, the all genes of each pathway are also used to directly group samples to evaluate the significance of detected subtype-factors.

Obviously, the above analysis routine can be applied on any other dataset of samples with different kinds of complex diseases.

Noted, our proof-of-concept study is to combine a group of genes with differential expression and a group of genes with differential expression variance. (i) We don’t select the genes with high variances, but the genes whose expression variances can distinguish different conditions/phenotypes. If the expression variance of a gene can classify samples with different phenotypes well, the dominant component of this gene’s variance could be biological variance. Obviously, the technique variance should have no such discrimination. (ii) We have also checked the correlation between the variance of each DEVG and each clinical index. Many genes’ expression variances even have significant correlation with clinics, which would not be caused by technique variance too. (iii) Our experiment on microarray is an application of IEA to support the idea of combining DEG and DEVG, and the results show our method’s efficiency. Indeed, many approaches are still proposing to improve the selection of conventional DEG or even new DEVG. The removal of technique variance will improve the selection of DEVG and final IEA, which is our future work.

#### Diabetes associated genes on pathways

First of all, we investigated the overlap between prior-known disease genes (e.g., diabetes associated genes) and pathway genes. Many pathways are full of diabetes associated genes (Additional file 6: Table S6), which means pathways could have great changes during disease development and progression. These pathways would be causes or outcomes of the disease. Although IEA pays attention to the identification of dysfunctional pathways (e.g., subtype-relevant pathways) rather than discrimination of causal pathways, as introduced in follows, the map of pathways can further complementally supply some clues of the causal roles of pathways at the level of network of networks [57].

Then on the main dataset GSE41762 [52], we have obtained feature genes as summarized in Table 4. There are 2558 DEGs and 345 DEVGs selected by IEA, many of them are also detected on the replicate dataset GSE38642 [53]. The 523 genes of DEGs are disease genes, and 658 genes are pathway genes; meanwhile, the 63 genes of DEVGs are disease genes and 79 genes are pathway genes. Obviously, there are many disease-informative or function-informative genes disregarded in conventional analysis (i.e., DEVGs rather than DEGs), and IEA can capture these genes and estimate their effects in the dysfunction of pathways.

**Table 4:** The statistic on DEGs, DEVGs and their overlapping with pathway or disease genes (Diabetes)

	DEG^a^	DEVG	PG_DEG	DG_DEG	PG_DEVG	DG_DEVG	DEGup	DEGdown	DEVGup	DEVGdown
GSE41762	2558	345	658	523	79	63	1493	1065	160	185
GSE38642	2306	632	647	515	167	128	1294	1012	389	243
Overlapping	836	28	246	10	219	3	489	346	15	13
Significance	0	7.4832e-07	0	3.3539e-11	0	0.00059927	0	0	5.2401e-08	3.6083e-08

^a^DEG points genes with differential expression; DEVG points genes with differential expression variance; PG_DEG points the pathway genes in DEGs, i.e., the overlaps between pathway genes and DEGs; DG_DEG points the disease genes in DEGs, i.e., the overlaps between disease genes and DEGs; PG_DEVG points the pathway genes in DEVGs, i.e., the overlaps between pathway genes and DEVGs; DG_DEVG points the disease genes in DEVGs, i.e., the overlaps between disease genes and DEVGs; DEGup and DEGdown point genes with up-regulation and down-regulation respectively; DEVGup and DEVGdown point genes with relax-regulation and tight-regulation respectively

**Fig. 4:**
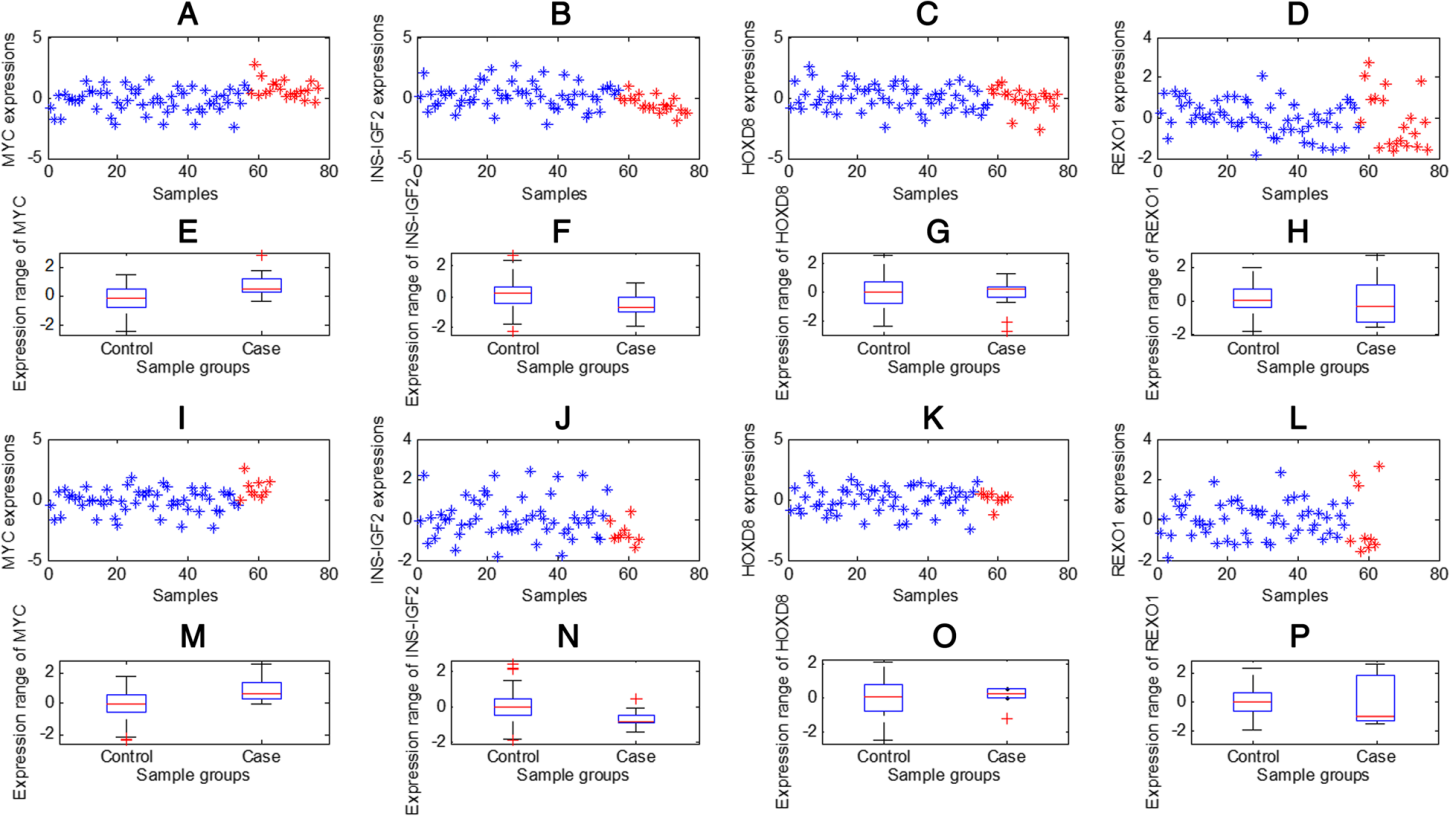
The cases of differential expression patterns of DEG and DEVG. For genes MYC, INS-IGF2, HOXD8, and REXO1, (**a**)-(**d**) give their expression profiles on dataset GSE41762; (**e**)-(**h**) give their expression distribution on dataset GSE41762; (**i**)-(**l**) give their expression profiles on dataset GSE38642; (**m**)-(**p**) give their expression distribution on dataset GSE38642

Furthermore, in DEGs, there are 1493 gene up-regulated in disease state and 1065 genes down-regulated. Meanwhile, there are 185 genes tight-regulated in disease condition and 160 genes relax-regulated. The examples of such four expression patterns are shown in Fig. 4. MYC, known as a cancer oncogene, is also reported to be altered in diabetes [58]. Seeing Fig. 4a, MYC is indeed up-regulated in the diabetes state. Insulin is known as a main cause of diabetes [59], and its an isoform as INS-IGF2 actually has down-regulation when diabetes occurs (Fig. 4b). HOXD8, as a gene in the homeobox family encoding a highly conserved family of transcription factors, has an important role in the morphogenesis. It has tight-regulation in diabetes condition (Fig. 4c), so that, it may participate in the accurately regulation [21] of biological processes associated to diabetes. By contrast, REXO1, known as Transcription elongation factor B polypeptide 3-binding protein 1, would be a cofactor involved in gene regulation [60]. This gene shows relax-regulation in diabetes condition (Fig. 4d). Thus, REXO1 would be a cause or indicator of some subtypes of diabetes. Indeed, the original study has supplied four clinical indices [52], we found *age* is mostly related to the subtypes or sample clusters of diabetes determined by REXO1 (Seeing Additional file 11: Table S11 and Additional file 12: Table S12).

More importantly, DEVGs (either "tight" or "relax" expression) don’t mean no-changes. As the key point of our model and method, DEVG means a gene would denote activation of a signalling pathway (or sub-pathway) in a group of samples, meanwhile, inactivation of this signalling pathway (or sub-pathway) in another group of samples. This would be a main cause of heterogeneous samples. The biological mechanism underlying this phenomenon would be the switch of pathway activation. If based on other kinds of enrichment analysis framework, it is possible to discuss the activation, inactivation, or activation-switch of a signalling pathway, which will be studied in our future work.

#### Dysregulated pathways identified to capture DEGs and DEVGs simultaneously

**Fig. 5:**
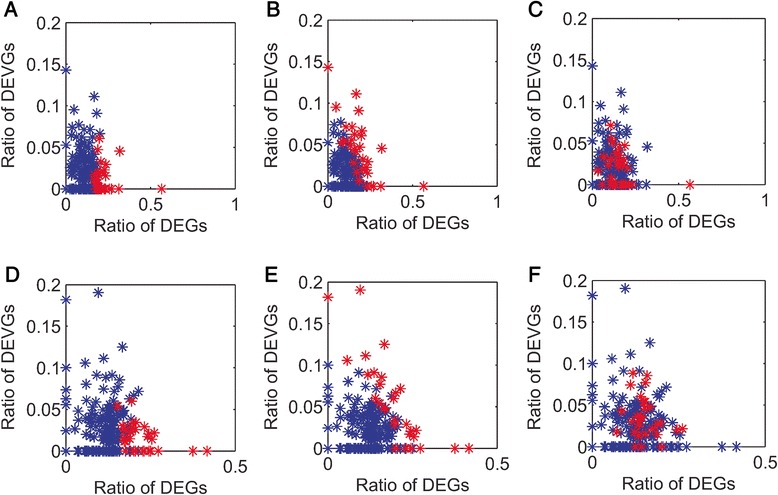
The gene-distribution graph of pathways identified by different methods. **a**-**c** The pathways high-ranked (labelled in red) by ORA, IEA and GSEA respectively, whose results on dataset GSE41762. **d**-**f** The pathways high-ranked by ORA, IEA and GSEA respectively, whose results on dataset GSE38642

In conventional analysis, the genes with differential expression are focused; now, the genes with differential expression variance are also attractive. In the context of differential expression variance, the dysregulated pathways are expected to have as many DEGs & DEVGs as possible, which can be captured by over-representation approach like the proposed IEA. For evaluation, a gene-distribution graph is further introduced to show the percentages of DEGs and DEVGs respectively for each pathway. In Fig. 5, a pathway is represented by a point whose indices in axis are the percentages of DEGs and DEVGs in this pathway respectively. Obviously, the pathways full of DEGs and DEVGs tend to locate at the right-up conner of such gene-distribution graph.

To validate the pathways identified by IEA and compare to other methods ORA and GSEA, we firstly calculated the percentages of DEGs and DEVGs of each KEGG pathway and plot them on the gene-distribution graph. Then we calculated the dysfunctional score (i.e., enrichment) and rank all KEGG pathways, and found the Top-30 selected pathway (labelled in red) in graph. Obviously, the pathways high-ranked by ORA have least scores located at the right-bottom of gene-distribution graph (Fig. 5a). It means the pathways selected by ORA are full of DEGs rather than DEVGs. By contrast, in the IEA, the detected pathways are full of DEGs and DEVGs respectively and significantly (Fig. 5b). Even more, the dysregulated pathways detected by well-known GSEA [1] on these datasets shown weak performance on the identification of pathways full of DEVGs (Fig. 5c). Thus, IEA indeed can effectively detect the pathways under-scored in conventional analysis, and these dysfunctional pathways would be disease-relevant or subtype-relevant. This conclusion is also supported by the similar results from the analysis on replicate dataset (Fig. 5d-f).

Noted, the pathways identified by different methods can be significantly observed in the analysis on the replicated dataset (*P*-value less than 0.05, whose details are supplied in Additional file 7: Table S7). The issue of the consensus of pathway identification is not discussed more here, although some other studies have worked to improve the robustness of dysregulated pathway identification by integrating other prior information (e.g., biological network or GO annotation) [16].

In the high-ranked pathways identified by IEA, many pathways are actually full of DEVGs. These DEVGs could be further associated to potential subtypes of samples, which are discussed in follows. Besides, some of these pathways under-scored by other methods indeed have been reported to be altered in the disease state (e.g., diabetes). For examples,

(i) 'KEGG HEMATOPOIETIC CELL LINEAGE'. Diabetes is known to compromise the function of the bone marrow (BM) [61], and diabetic complications mainly including macrovascular events might be from the dysfunctional BM-derived hematopoietic cells.
(ii) 'KEGG CYTOKINE-CYTOKINE RECEPTOR INTERACTION'. Cytokines regulate inflammatory and immune responses, which play important roles in the pathogenesis of diabetes and its microvascular complications. The functional variations of cytokines and their receptors can benefit the prediction of the susceptibility and progression to Diabetic nephropathy (DN) [62]. As the potential pathogenic mediators in DN, cytokines might provide new potential therapeutic agents for disease treatments.

#### Dysregulated pathway-crosstalk identified to reveal the interactive map and module among pathways

Different from conventional strategy to use the overlapping genes as pathway crosstalks [27], two-way RWR is assistantly applied to find the interactive genes between any two pathways. RWR is previously used in the rank of disease genes [28], which holds the assumption that the candidate pathogen genes are more proximate to the known disease genes than randomly selected genes. This assumption is expected to be hold for pathway genes too.

We first evaluated the pathway genes possibly selected by RWR,which uses the identified DEGs & DEVGs in a pathway as seeds. In the high-scored genes by RWR, there is a significant amount of pathway genes (seeing Table 5), which is the same as disease genes ranked by RWR (Table 6). In the evaluation in Table 5, we have set two kinds of control experiments. One is the prior-known network used, i.e., two sources as STRING [38] and HPRD [63] are both applied; the other one is the feature genes used in the given background network, i.e., three kinds of feature genes (as all ranked genes with *P*-values, the ranked DEGs with *P*-values and the ranked genes from RWR excluding the seeds) are respectively used to calculate the AUC values [28] to evaluate the efficiency of selecting/ranking pathway genes or disease-associated genes. Depending on these experiments, we can find:

**Table 5:** The AUC of different rank lists for pathway genes (Diabetes)

PIN	STRING-based		HPRD-based	
Data	GSE41762	GSE38642	GSE41762	GSE38642
All genes	0.4861^a^	0.48611	0.4861^a^	0.48611
DEGs	0.46342	0.51717	0.46342	0.51717
twRWR	0.83498	0.83326	0.68449	0.68023

^a^For feature genes like all genes and DEGs, they don’t use network information, so that, they have the same AUC values on the same dataset although different network used

**Table 6:** The AUC of different rank lists for disease-associated genes (Diabetes)

PIN	STRING-based		HPRD-based	
Data	GSE41762	GSE38642	GSE41762	GSE38642
All genes	0.46994^a^	0.46567	0.46994^a^	0.46567
DEGs	0.45695	0.47277	0.45695	0.47277
twRWR	0.73546	0.73652	0.70633	0.69904

^a^For feature genes like all genes and DEGs, they don’t use network information, so that, they have the same AUC values on the same dataset although different network used

(i) As a control, when all genes are ranked according to *P*-values, its AUC is low. And when the selected DEGs are ranked according to *P*-values, the AUC is similar to that of all genes. Meanwhile, when the genes from two-way RWR excluding seeds are ranked according to proximity values, the AUC achieves highest, which support again RWR-based approach is effective to capture interactive phenotypic genes as pathway genes or disease-associated genes. Thus, two-way RWR is effective to mimic the pathway crosstalk and construct the associations among pathways, which is obviously consistent in multiple control experiments by using different prior-known network (e.g., STRING and HPRD), or different datasets (e.g., GSE41762 and GSE38642), or even different ranked/selected feature genes (e.g., pathway genes and disease-associated genes).
(ii) The protein association network (as in STRING [38]) rather than protein physical network (as in HPRD [63]) would be more efficient to lead the two-way RWR to link seed genes to pathway genes or disease-associated genes. There are at least two reasons for this result: one is that the known protein physical network is still greatly incomplete, by contrast protein association network would supply additional predicted interactions with high confidence; the other one is that, except for direct interactions between pathways, protein association network would cover much more indirect interactions or long-term interactions, which would mimic the pathway crosstalk well. In all, protein association network is efficient to detect the associations among pathways, whose usage to accurately predict physical interaction of pathways is out of this work and would be a future study.

**Fig. 6:**
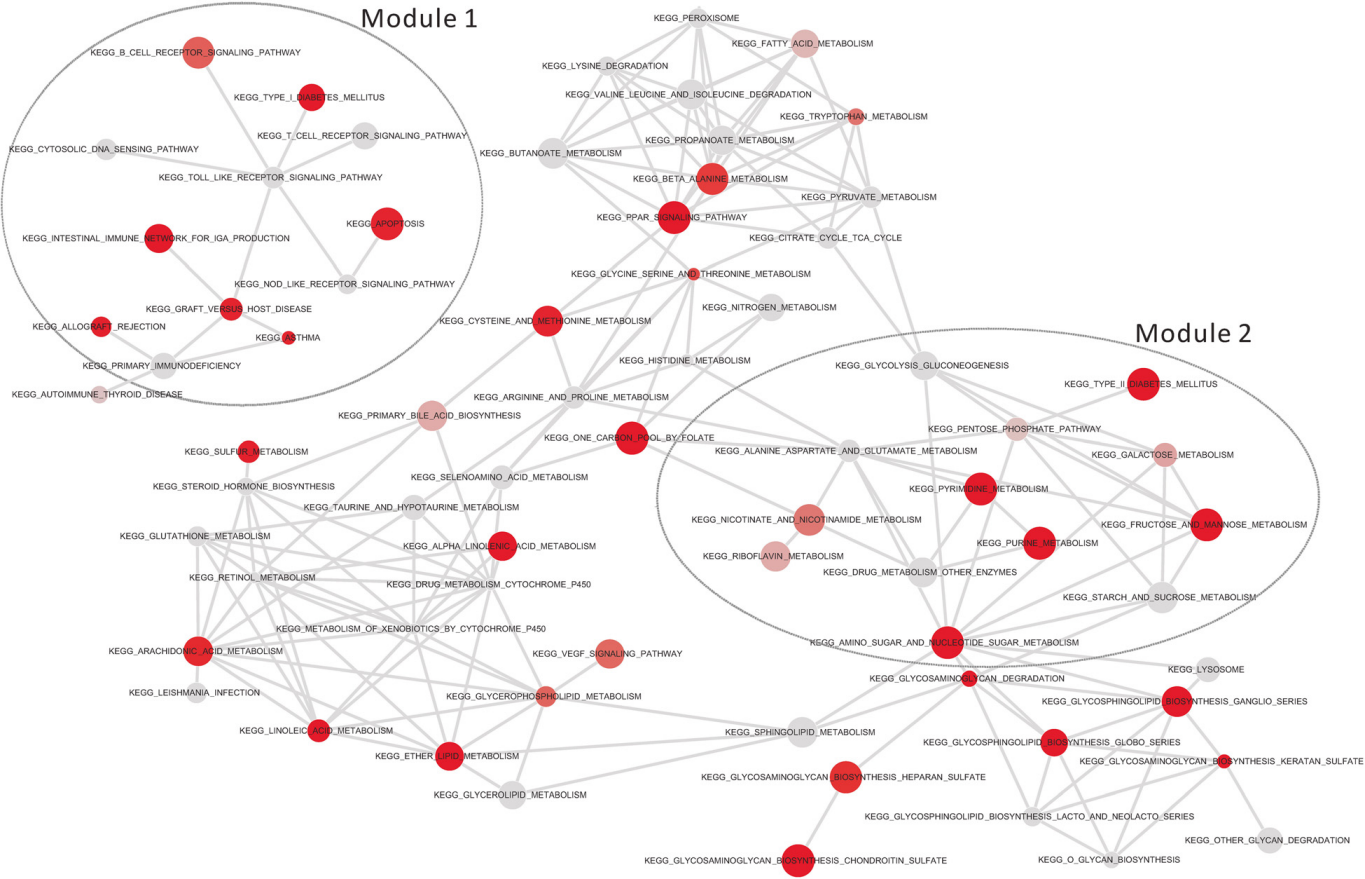
The main topological structure of the map of pathways reconstrcucted on diabetes datasets. Module 1 is full of signalling pathways, and Module 2 is full of disease/diabetes associated pathways

Thus, RWR is actually effective to detect the genes interactive within known pathway genes. Then by two-way RWR, we can find the interactive genes from two pathways, and select any pathway-pair as a crosstalk significantly (Additional file 8: Table S8). All the crosstalks connect the known pathways as a map, where each crosstalk is an edge and a pathway is a node. This map of pathways is a network of networks, rather than the original background network of separate molecules. In the map of pathways, the modules of interactive pathways can be detected, where a module represents a group of closely inter-connected pathways. In these pathway modules, two modules are obviously related to diabetes. One module (Module 1 shown in Fig. 6) is the group of signalling pathways. Signalling pathways are known as the up-stream functions in the cascades of signals, so that, they have great possibility to be the causes of the dysfunction of down-stream functions, e.g., diabetes pathways. The other module (Module 2 shown in Fig. 6) is just the group of pathways concerning glycolysis, sugar metabolism, glycosaminoglycan and diabetes, which seems to be extremely a core pathway module of diabetes. Noted, the Type II diabetes pathway would have significant crosstalk with pentose phosphate pathway as shown in Fig. 6. As reported, the pentose phosphate pathway is widely activated in diabetes and its complications [64–67], thus this pathway would be important to understand the risk of diabetes diagnosis and treatment in clinical application. Obviously, the pentose phosphate pathway have no significance on the selection by IEA or other methods, and actually, its importance is reflected from the topological structure of the map of pathways. These facts reveal: (i) conventional approaches usually focus on single pathways, so that, they can sometimes capture the disease associated pathways relevant to particular phenotypes but can’t distinguish or underestimate the potential causal relationship among pathways; (ii) the proposed IEA supplies the map of pathways to reflect the functional organization of pathways, and disclose the key modules of pathways, such as the upstream pathways related to signalling pathways and the downstream pathways associated to diseases; (iii) on the map of pathways, those pathways full of DEVGs and DEGs tend to interact with disease pathways, indicating the determinant of subtypes (i.e., the subtype-factors identified in follows) are actually also the potential determinants of diseases. Totally, the map of pathways supplies us a new viewpoint of functional organization at the level of network of networks.

#### Dysregulated pathways associated to clinical indicator as subtype-factors

The pathways full of DEGs and DEVGs are specially selected by IEA, in which the DEVGs might be the cause of potential subtypes of samples. Compared to the mean values of genes in case, the samples in control can be divided into two groups: one group of samples have larger gene expressions than that mean value; and the other group of samples have fewer values, or vice versa. Thus, we have applied two strategies to cluster the samples in control or case group, which can associate one pathway to some clinical index. The first common strategy (noted as PGC) is using the expressions of whole genes in a pathway to cluster samples into two clusters, and test the significance of these two groups of samples on one clinical indicator (The test is to see if one group of samples have larger or fewer clinical values than those of the other group of samples). The second strategy (just as proposed SLC) is using the discrete value of DEVGs in the same pathway to group samples and measuring their relationships with clinical indices.

**Fig. 7:**
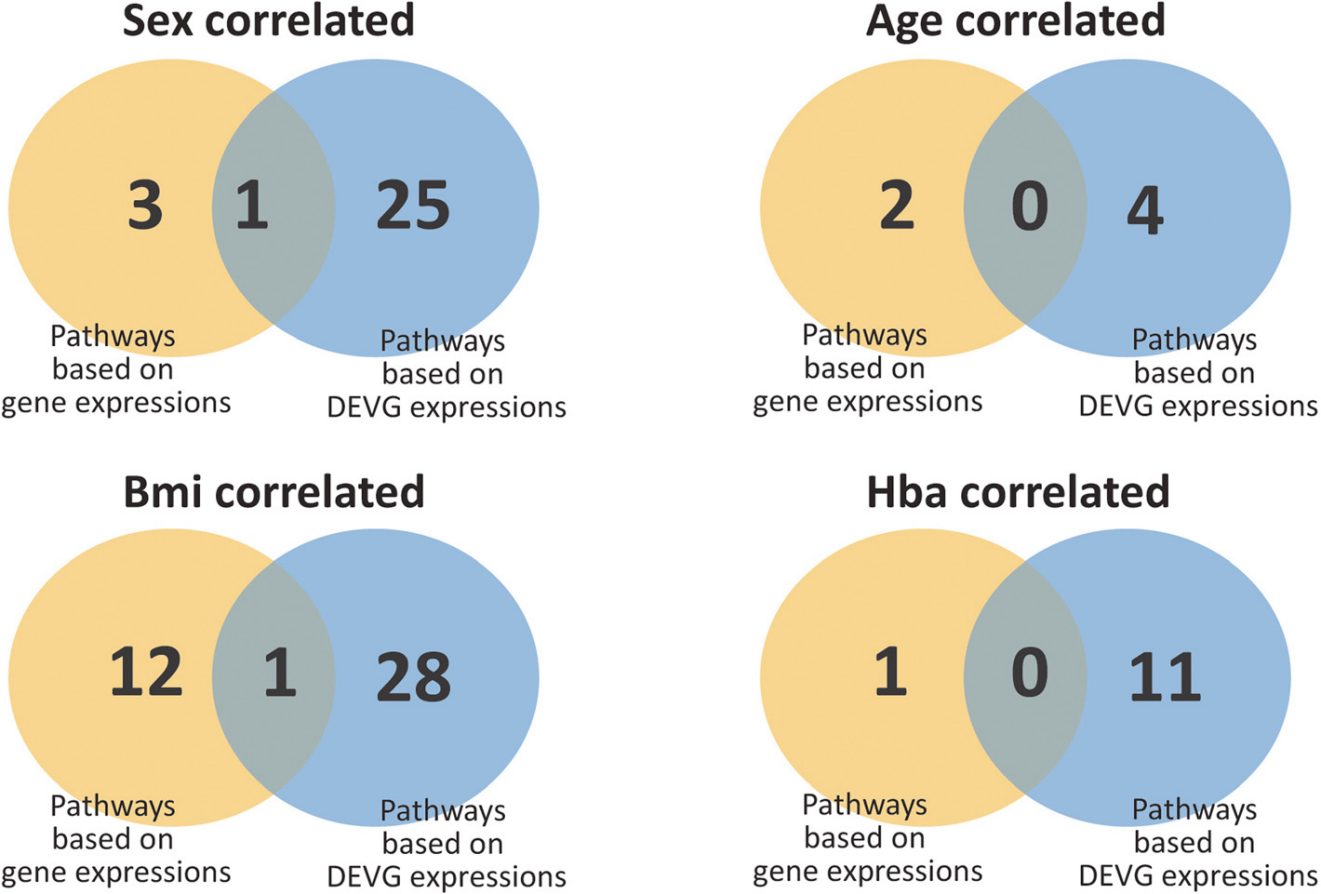
The summary of dysregulated pathways identified as subtype-factors corresponding to four clinical indices. For example, there are 11 pathways identified by IEA to be correlated with Hba values (i.e., based on DEVG expressions of pathway genes), but, there are only 1 pathways recognized by conventional strategy (i.e., bsaed on raw expressions of pathway genes). Similarly, for other clinical indices, IEA can also identify many subtype-factors

Additional file 9: Table S9 gives the *P* value of the association of each pair between a pathway and a clinic index for normal samples, and Additional file 10: Table S10 gives those for diabetes. Generally, SLC tends to discover more significant potential subtypes of samples corresponding to particular clinic index (Fig. 7). Thus, DEVGs actually have more power to identify the subtypes of genotype-phenotype associations than conventional approaches based on differential expression only.

Obviously, one pathway can associate to multiple clinical indices, and one clinical index can also relate to multiple pathways. ‘KEGG WNT SIGNALING PATHWAY’ is consistently related to bmi in the analysis of duplicate datasets. In fact, this pathway already has a few evidences on their associations with body weight [68–70]. ‘KEGG CELL CYCLE’ is found to possibly associate with age, sex, bmi or Hba, which is known as a common factor in disease development and progression [71].

Although each DEVG can separately determine some potential subtype-factor, here, we only discuss the determination of DEVG combination at the level of pathway. In addition, the association of each pair between a gene (i.e., one DEVG) and a clinical index are listed in Additional file 11: Table S11 and Additional file 12: Table S12 for normal and diseased samples respectively.

Noted, subtype is a potential biological explanations of DEVG, so that, we have evaluated the possible subtype-factors rather than subtypes by the association between DEVG (or DEVG-full pathways) and known clinical index. In the statistical analysis, we have analyzed age, sex, bmi, and even Hba1c which evaluates the risk of diabetes. Thus, this clinical information can help us to find subtype of genotype-phenotype associations as stated above. Of course, in breast cancer, there are some well-known subtypes determined on genotypes. To the best of our knowledge, in diabetes, the subtypes as T1D, T2D, Gestational diabetes, Surgically induced diabetes, Chemically induced diabetes, are not defined by one or two genes/proteins. Thus, our finding of subtype-factors (DEVGs or pathways) would be the causes or indicators of disease (e.g., diabetes) subtypes on genome level, which will be further studied in future.

## Conclusion

Pathway enrichment analysis is a useful tool in the study of biology or biomedicine, due to its functional screening on the well-known biological processes rather than single molecules. The measurement of dysfunctions of pathways during a phenotype change, e.g., from normal to diseased, is the key issue when applying enrichment analysis for pathway or other functional gene set. Different from differentially expressed genes focused in previous methods, the genes with great differential expression variance are also attractive and important, which indicate another specific characteristic of a biological system in the change of phenotypes.

In the context of differential expression and expression variance, IEA is proposed to identify the pathways full of DEGs and DEVGs simultaneously, rather than conventional approaches focusing on only DEGs. The biological meaning of IEA has obtained strong evidences by an evaluation scheme based on method comparison. On the real datasets of disease samples, IEA indeed specifically identify pathways containing DEGs and DEVGs, which are usually under-scored by other methods. The map of pathways was further reconstructed based on the selected pathway crosstalks, and the module organization among pathways was also detected. The topological structure of such network of pathways reveals the signalling pathways as upstream functions would be causes of disease, and the disease-relevant pathways as downstream functions would link to those upstream pathways by crosstalk. In addition, some disease-relevant pathways or subtype-relevant pathways are well associated with clinical indices according to their DEVGs’ relative expression level, which are usually not observed from the raw expression profiles of pathway genes. Although many identified subtype-factors haven’t clinical evidences due to the limit in the clinical application, the IEA actually show its ability to identify the risk of subtypes of genotype-phenotype associations. Those subtype-factors could help us in accurately realizing personal prevention or personal treatment [72, 73]. Besides, the additional analysis results on colorectal cancer also support these conclusions (Additional file 13: SI document - a case study on colorectal cancer and Table A1-A3; Additional file 14: Table A4; Additional file 15: Table A5).

Totally, IEA supplies a new way to carry on enrichment analysis in the context of differential expression and expression variance, and can easily expand to handle with the analysis in other more complicated context (e.g., the differential expression covariance). It is also necessary to expand IEA to functional class scoring or pathway topology based approaches in future work.

## Additional files

Additional file 1: Table S1. The 3-order approach-specific datasets corresponding to different methods based on prioritization performance. (DOCX 16 kb) Additional file 2: Table S2. The prioritization performance about method comparison on approach-specific datasets (K = 2). (DOCX 17 kb) Additional file 3: Table S3. The prioritization performance about method comparison on approach-specific datasets (K = 4). (DOCX 17 kb) Additional file 4: Table S4. The sensitivity performance about method comparison on approach-specific datasets (K = 4). (DOCX 17 kb) Additional file 5: Table S5. The 3-order approach-specific datasets corresponding to different methods based on sensitivity performance. (DOCX 16 kb) Additional file 6: Table S6. Overlaps between pathway genes and disease associated genes on diabetes datasets. (XLS 39 kb) Additional file 7: Table S7. Pathway ranking by different methods on diabetes datasets. (XLSX 22 kb) Additional file 8: Table S8. Pathway-pair as a crosstalk identified on diabetes datasets. (XLS 107 kb) Additional file 9: Table S9. Association of each pair between a pathway and a clinic index (for Normal samples) on diabetes datasets. (XLS 56 kb) Additional file 10: Table S10. Association of each pair between a pathway and a clinic index (for Diabetes samples) on diabetes datasets. (XLS 60 kb) Additional file 11: Table S11. Association of each pair between a DEVG and a clinic index (for Normal samples) on diabetes datasets. (XLS 96 kb) Additional file 12: Table S12. Association of each pair between a DEVG and a clinic index (for Diabetes samples) on diabetes datasets. (XLS 119 kb) Additional file 13:
**SI document - a case study on colorectal cancer and Table A1-A3.** (DOCX 3072 kb) Additional file 14: Table A4. Pathways ranking for colorectal cancer. (XLS 97 kb) Additional file 15: Table A5. Pathway-pair as a crosstalk (colorectal cancer). (XLS 133 kb)

## Abbreviations

DEG: Differentially expressed gene
DEVG: Gene with great differential expression variance
IEA: Integrative enrichment analysis
GSEA: Gene set enrichment analysis
RWR: Random walk with restart
SLC: Supervised-like clustering approach
PIN: Protein interaction network
