# Supplementary material for: Integrative enrichment analysis: a new computational method to detect dysregulated pathways in heterogeneous samples
Source: BMC Genomics. 2015 Nov 10;16:918. doi: 10.1186/s12864-015-2188-7 (PMC4641376; doi:10.1186/s12864-015-2188-7)
Supplement: Additional file 1: Table S1. — The 3-order approach-specific datasets corresponding to different methods based on prioritization performance. (DOCX 16 kb) [file 12864_2015_2188_MOESM1_ESM.docx]

**Table S1 The 3-order approach-specific datasets corresponding to different methods based on prioritization performance**

| ID | Approach-specific datasets |
| --- | --- |
| **GSA-specific** | GSE1297，GSE14762，GSE14924_CD4，GSE14924_CD8，GSE15471，GSE16515，GSE19728，GSE20153，GSE20291，GSE24739_G1，GSE32676，GSE5281_EC，GSE5281_HIP，GSE5281_VCX，GSE6956AA，GSE6956C，GSE7305，GSE8671，GSE8762，GSE9476 |
| **PADOG-specific** | GSE1297，GSE14762，GSE14924_CD4，GSE14924_CD8，GSE15471，GSE16515，GSE16759，GSE18842，GSE19188，GSE19420，GSE19728，GSE21354，GSE23878，GSE24739_G0，GSE24739_G1，GSE32676，GSE3467，GSE3678，GSE4183，GSE5281_HIP，GSE5281_VCX，GSE6956C，GSE7305，GSE8671，GSE8762，GSE9348，GSE9476 |
| **IEA-specific** | GSE3467，GSE3585，GSE4107，GSE4183，GSE6956AA，GSE781 |
| **MRGSE-specific** | GSE1145，GSE14924_CD4，GSE14924_CD8，GSE18842，GSE19420，GSE19728，GSE20153，GSE20164，GSE23878，GSE3678，GSE5281_HIP，GSE5281_VCX |
| **ORA-specific** | GSE14762，GSE16759，GSE19188，GSE3467，GSE3585，GSE4183，GSE5281_EC，GSE7305，GSE781 |
| **GLOBALTEST-specific** | GSE1145，GSE19420，GSE20164，GSE21354，GSE24739_G0，GSE3585，GSE3678，GSE4107，GSE6956C，GSE8671，GSE9348，GSE9476 |
| **GSVA-specific** | GSE1297，GSE15471，GSE16515，GSE16759，GSE19188，GSE20164，GSE20291，GSE24739_G1，GSE5281_EC，GSE6956AA |
| **PLAGE-specific** | GSE1145，GSE18842，GSE20153，GSE20291，GSE21354，GSE23878，GSE24739_G0，GSE32676，GSE4107，GSE781，GSE8762，GSE9348 |
