# Supplementary material for: Integrative enrichment analysis: a new computational method to detect dysregulated pathways in heterogeneous samples
Source: BMC Genomics. 2015 Nov 10;16:918. doi: 10.1186/s12864-015-2188-7 (PMC4641376; doi:10.1186/s12864-015-2188-7)
Supplement: Additional file 2: Table S2. — The prioritization performance about method comparison on approach-specific datasets (K = 2). (DOCX 17 kb) [file 12864_2015_2188_MOESM2_ESM.docx]

**Table S2 The prioritization performance about method comparison on approach-specific datasets (K=2)**

| ID | **GSA**  **-specific** | **PADOG**  **-specific** | **IEA**  **-specific** | **MRGSE**  **-specific** | **ORA**  **-specific** | **GLOBALTEST**  **-specific** | **GSVA**  **-specific** | **PLAGE**  **-specific** |
| --- | --- | --- | --- | --- | --- | --- | --- | --- |
| **GSA** | **(4.70,2.84)** | (27.27,25.92) | (40.27,34.72) | (27.43,20.06) | (37.94,22.56) | (37.17,32.33) | (29.58,29.62) | (38.05,21.17) |
| **PADOG** | ***(4.37,2.79)*** | **(10.28,9.67)** | (25.97,25.12) | (21.50,17.25) | (27.50,22.04) | (19.85,22.52) | (20.14,12.78) | (26.62,22.02) |
| **IEA** | (41.71,26.67) | (51.99,26.23) | **(17.85,14.78)** | (66.97,18.27) | (34.65,23.31) | (45.84,26.37) | (48.82,32.35) | (58.75,32.96) |
| **MRGSE** | (47.27,27.44) | (53.50,28.52) | (56.49,34.19) | **(18.33,13.40)** | (83.82,12.55) | (46.96,30.00) | (52.36,26.64) | (61.91,23.17) |
| **ORA** | (45.42,33.67) | (48.33,27.43) | (30.26,15.44) | (69.93,21.97) | **(14.01,7.30)** | (53.58,25.05) | (36.86,29.30) | (55.54,28.52) |
| **GLOBALTEST** | (28.16,13.45) | (31.05,19.60) | (29.29,20.08) | (36.73,22.93) | (41.40,23.93) | **(15.60,17.66)** | (51.67,17.63) | (41.96,21.61) |
| **GSVA** | (23.30,15.57) | (50.08,28.53) | (57.72,33.36) | (41.15,23.68) | (61.01,29.74) | (66.11,28.34) | **(16.01,11.41)** | (53.81,27.08) |
| **PLAGE** | (19.48,11.15) | (30.61,20.49) | (42.18,34.22) | (33.84,19.36) | (43.04,30.18) | (33.83,28.26) | (31.40,19.99) | **(15.72,11.95)** |
