# Supplementary material for: Integrative enrichment analysis: a new computational method to detect dysregulated pathways in heterogeneous samples
Source: BMC Genomics. 2015 Nov 10;16:918. doi: 10.1186/s12864-015-2188-7 (PMC4641376; doi:10.1186/s12864-015-2188-7)
Supplement: Additional file 3: Table S3. — The prioritization performance about method comparison on approach-specific datasets (K = 4). (DOCX 17 kb) [file 12864_2015_2188_MOESM3_ESM.docx]

**Table S3 The prioritization performance about method comparison on approach-specific datasets (K=4)**

| ID | **GSA**  **-specific** | **PADOG**  **-specific** | **IEA**  **-specific** | **MRGSE**  **-specific** | **ORA**  **-specific** | **GLOBALTEST**  **-specific** | **GSVA**  **-specific** | **PLAGE**  **-specific** |
| --- | --- | --- | --- | --- | --- | --- | --- | --- |
| **GSA** | **(14.89,12.16)** | (26.72,24.50) | (39.13,30.33) | (40.08,27.21) | (36.28,30.58) | (32.65,28.81) | (24.10,23.48) | (32.24,28.63) |
| **PADOG** | ***(11.40,11.97)*** | **(13.52,13.44)** | (25.18,24.25) | ***(21.26,19.28)*** | (21.58,20.06) | ***(15.47,20.33)*** | ***(15.78,11.22)*** | ***(16.51,16.94)*** |
| **IEA** | (48.92,28.11) | (51.84,26.92) | **(17.93,10.83)** | (65.29,19.55) | (33.74,15.95) | (40.67,25.61) | (58.40,28.00) | (54.78,28.63) |
| **MRGSE** | (52.06,28.94) | (50.91,29.59) | (66.63,28.97) | **(25.77,16.79)** | (69.11,24.65) | (55.61,28.92) | (45.89,27.18) | (46.36,27.90) |
| **ORA** | (45.87,29.94) | (49.43,28.20) | (29.89,19.24) | (69.29,17.72) | **(20.77,13.57)** | (43.03,27.44) | (44.01,27.20) | (57.78,25.60) |
| **GLOBALTEST** | (33.99,22.16) | (32.10,21.68) | (27.95,15.78) | (34.02,18.80) | (37.95,19.30) | **(19.07,15.71)** | (47.56,20.07) | (30.87,19.68) |
| **GSVA** | (36.80,27.60) | (47.11,29.58) | (53.19,28.78) | (53.63,25.42) | (43.90,27.58) | (63.90,26.41) | **(20.26,15.75)** | (52.59,29.10) |
| **PLAGE** | (25.32,17.32) | (28.75,20.07) | (38.78,32.44) | (37.18,24.53) | (43.35,21.78) | (29.15,25.41) | (34.68,18.65) | **(20.96,17.26)** |
