# Supplementary material for: Integrative enrichment analysis: a new computational method to detect dysregulated pathways in heterogeneous samples
Source: BMC Genomics. 2015 Nov 10;16:918. doi: 10.1186/s12864-015-2188-7 (PMC4641376; doi:10.1186/s12864-015-2188-7)
Supplement: Additional file 4: Table S4. — The sensitivity performance about method comparison on approach-specific datasets (K = 4). (DOCX 17 kb) [file 12864_2015_2188_MOESM4_ESM.docx]

**Table S4 The sensitivity performance about method comparison on approach-specific datasets (K=4)**

| ID | **GSA**  **-specific** | **PADOG**  **-specific** | **IEA**  **-specific** | **MRGSE**  **-specific** | **ORA**  **-specific** | **GLOBALTEST**  **-specific** | **GSVA**  **-specific** | **PLAGE**  **-specific** |
| --- | --- | --- | --- | --- | --- | --- | --- | --- |
| **GSA** | **(0.084,0.10)** | (0.18,0.23) | (0.29,0.28) | (0.31,0.25) | (0.55,0.31) | (0.25,0.26) | (0.17,0.21) | (0.22,0.23) |
| **PADOG** | (0.093,0.13) | **(0.065,0.095)** | (0.18,0.18) | (0.20,0.20) | (0.38,0.20) | (0.14,0.17) | (0.12,0.11) | (0.13,0.17) |
| **IEA** | (0.13,0.18) | (0.12,0.12) | **(0.056,0.071)** | ***(0.12,0.095)*** | ***(0.037,0.057)*** | (0.098,0.12) | (0.11,0.13) | (0.095,0.12) |
| **MRGSE** | (0.64,0.29) | (0.51,0.31) | (0.54,0.29) | **(0.13,0.12)** | (0.69,0.20) | (0.48,0.32) | (0.41,0.31) | (0.51,0.31) |
| **ORA** | (0.33,0.32) | (0.45,0.29) | (0.29,0.23) | (0.52,0.33) | **(0.16,0.13)** | (0.38,0.29) | (0.37,0.29) | (0.37,0.29) |
| **GLOBALTEST** | ***(0.081,0.12)*** | ***(0.017,0.050)*** | ***(0.032,0.069)*** | ***(0.048,0.11)*** | ***(0.048,0.064)*** | **(0.019,0.062)** | (0.033,0.080) | ***(0.030,0.074)*** |
| **GSVA** | (0.16,0.19) | (0.26,0.28) | (0.29,0.27) | (0.25,0.21) | (0.28,0.24) | (0.22,0.26) | **(0.018,0.024)** | (0.21,0.24) |
| **PLAGE** | ***(0.069,0.10)*** | ***(0.039,0.13)*** | (0.059,0.13) | ***(0.049,0.099)*** | (0.19,0.24) | (0.041,0.12) | (0.029,0.071) | **(0.033,0.077)** |
