# Supplementary material for: Integrative enrichment analysis: a new computational method to detect dysregulated pathways in heterogeneous samples
Source: BMC Genomics. 2015 Nov 10;16:918. doi: 10.1186/s12864-015-2188-7 (PMC4641376; doi:10.1186/s12864-015-2188-7)
Supplement: Additional file 13: — SI document - a case study on colorectal cancer and Table A1-A3. (DOCX 3072 kb) [file 12864_2015_2188_MOESM13_ESM.docx]

**Supplementary of**

**Integrative enrichment analysis: a new computational method to detect dysregulated pathways in heterogeneous samples**

Xiangtian Yu^1^, Tao Zeng^2,*^, Guojun Li^1,*^

^1^ School of Mathematics, Shandong University, Jinan 250100, China

^2^ Key Laboratory of Systems Biology, Institute of Biochemistry and Cell Biology, Shanghai Institutes for Biological Sciences, Chinese Academy of Sciences, Shanghai 200031, China

**A1 Framework of IEA on transcriptional analysis of complex diseases (colorectal cancer)**

Firstly, data needed in IEA have been prepared from the public resources: The gene expression data of colorectal tumors were downloaded through GEO; there are two datasets, the main dataset GSE25070 [[1](#_ENREF_1)] contains 26 samples with 18631 genes respectively from colorectal tumors and matched adjacent non-tumor colorectal tissues, and the replicate dataset GSE8671 [[2](#_ENREF_2)] contains 32 samples with 21213 genes respectively from collected adenomas with those of normal mucosa; the gene lists of 186 KEGG pathways are obtained from GSEA package; the human protein interaction network (PIN) are extracted from STRING database with confidence score no less than 0.9; meanwhile, colorectal cancer associated genes are searched from GeneCards database.

Secondly, different scores of pathway enrichment are calculated: (i) the conventional score (ORA) as P-values of hypergeometric distribution of DEGs in a pathway; (ii) the conventional score (GSEA) as P-values of estimated pathway enrichment; (iii) the new score (IEA) as P-values of hypergeometric distribution of differential genes (integrating DEGs and DEVGs) in a pathway calculated by proposed HT2 approach. For ORA or IEA, the thresholds of P-value of significance test on DEGs or DEVGs are both set as 0.05, and adopted a pervious strategy to select those feature genes: 1) select all genes with FDR adjusted p-values no more than 0.1; 2) if the genes selected are less than 200, re-select all genes with P-values no more than 0.05 and fold-change no less than1.5; 3) if the genes selected are still less than 200, directly use the top 1% of genes ranked by P-values.

Thirdly, pathway crosstalks are evaluated by two-way RWR approach. The interactions selected from PIN consist of differential network, where the selected interactions have significant correlation difference between normal and cancer groups. On this differential network, two-way RWR approach is used to find the pathway crosstalks. The most significant pathway crosstalks (the threshold of P-value of significance test is set as 0.001 strictly) consist of the map of pathways. Besides, the enrichments of pathway genes or disease-associated genes in the high-ranked genes of RWR are also analysed and evaluated by AUC.

Fourthly, the DEVGs in each pathway are used to group samples in two clusters by SLC approach, although there are not clinical index supplied for further evaluation.

**Table A1 The statistic on DEGs, DEVGs and their overlapping with pathway or disease genes (Colorectal cancer)**

|  | DEG | DEVG | PG_DEG | DG_DEG | PG_DEVG | DG_DEVG | DEGup | DEGdown | DEVGup | DEVGdown |
| --- | --- | --- | --- | --- | --- | --- | --- | --- | --- | --- |
| GSE25070 | 5665 | 1248 | 1559 | 1181 | 309 | 236 | 2441 | 3224 | 112 | 1136 |
| GSE8671 | 9807 | 806 | 2676 | 1877 | 192 | 150 | 4994 | 4813 | 97 | 709 |
| Overlapping | 3281 | 67 | 1106 | 18 | 855 | 16 | 1421 | 1651 | 2 | 62 |
| Significance | 0 | 0.0017615 | 0 | 5.9693e-11 | 0 | 1.0006e-12 | 0 | 0 | 0.014751 | 5.6905e-05 |

*DEG points genes with differential expression; DEVG points genes with differential expression variance; PG_DEG points the pathway genes in DEGs, i.e. the overlaps between pathway genes and DEGs; DG_DEG points the disease genes in DEGs, i.e. the overlaps between disease genes and DEGs; PG_DEVG points the pathway genes in DEVGs, i.e. the overlaps between pathway genes and DEVGs; DG_DEVG points the disease genes in DEVGs, i.e. the overlaps between disease genes and DEVGs; DEGup and DEGdown point genes with up-regulation and down-regulation respectively; DEVGup and DEVGdown point genes with relax-regulation and tight-regulation respectively.

**A1.1 Colorectal cancer associated genes on pathways**

On the main dataset GSE25070 [[1](#_ENREF_1)], we have obtained feature genes as summarized in Table A1. There are 5665 DEGs and 1248 DEVGs selected by IEA, many of them are also detected on the replicate dataset GSE8671 [[2](#_ENREF_2)]. The 1181 genes of DEGs are disease genes, and 1559 genes are pathway genes; meanwhile, the 236 genes of DEVGs are disease genes and 309 genes are pathway genes. Obviously, there are many disease-informative or function-informative genes (i.e. DEVGs rather than DEGs) disregarded in conventional analysis, and IEA can capture these genes and estimate their effects in the dysfunction of pathways.

Furthermore, in DEGs, there are 2441 gene up-regulated in disease state and 3224 genes down-regulated. Meanwhile, there are 1136 genes tight-regulated in disease condition and 112 genes relax-regulated.


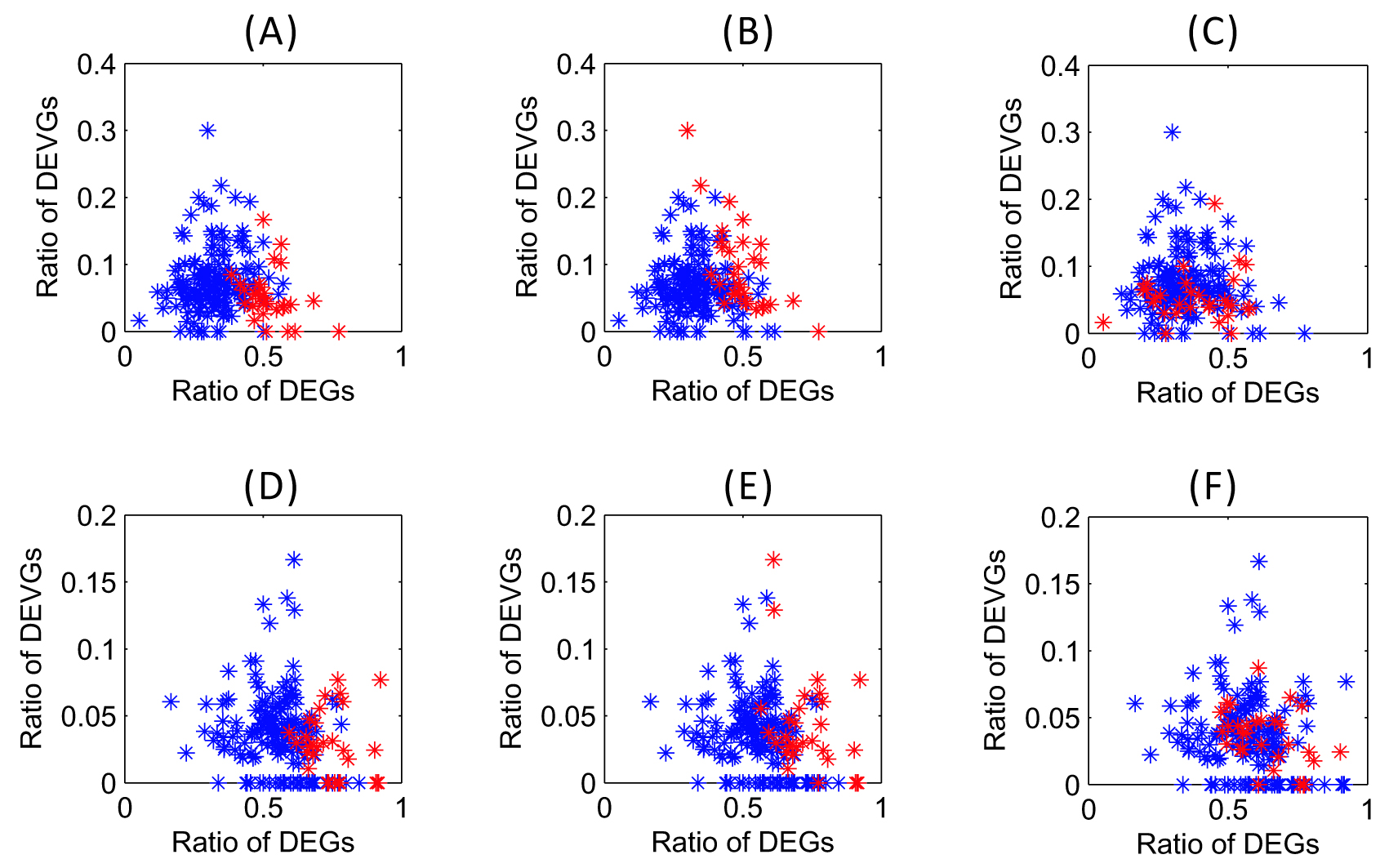


**Figure A1 The gene-distribution graph of pathways identified by different methods.** (A)-(C) The pathways high-ranked (labelled in red) by ORA, IEA and GSEA respectively, whose results on dataset GSE25070. (D)-(F) The pathways high-ranked by ORA, IEA and GSEA respectively, whose results on dataset GSE8671.

**A1.2 Dysregulated pathways identified to capture DEGs and DEVGs simultaneously**

Similar to the analysis on Diabetes datasets, we first calculated the percentages of DEGs and DEVGs of each KEGG pathway and plot them on the gene-distribution graph. Then we calculated the dysfunctional score (i.e. enrichment) of ORA and rank all KEGG pathways, and found the Top-30 selected pathway located at the right-bottom of gene-distribution graph (Figure A1 (A)). It means the pathways selected by ORA are full of DEGs rather than DEVGs. By contrast, the IEA calculated by HT2 can detect the pathways full of DEGs and DEVGs respectively and significantly (Figure A1 (B)). Again, the dysregulated pathways detected by GSEA on these datasets shown weak performance on the identification of pathways full of DEVGs (Figure A1 (C)). Thus, IEA indeed can effectively detect the pathways underscored in conventional analysis. This conclusion is also supported by the similar results from the analysis on replicate dataset (Figure A1 (D)-(F)). Of course, the pathways identified by different methods can be significantly observed in the analysis on the replicated dataset (P-value less than 0.05, whose details are supplied in Table A4).

In the high-ranked pathways identified by IEA, many pathways are actually full of DEVGs, some of which under-scored by other methods indeed have been reported to be altered in the disease state (e.g. colorectal tumors). For examples,

(i) ‘KEGG GLUTATHIONE METABOLISM’. Generally, the glutathione S-transferases (GST) are thought to be a personalized drug target for cancer treatment due to their specific expression pattern and active function in individuals [[3](#_ENREF_3)]. Compared to normal controls, GST activity is down-regulated in the patients at risk of colon cancer [[4](#_ENREF_4)], and particularly GSTT1 shown increased risk of colorectal cancer for individuals in a comprehensive review study [[5](#_ENREF_5), [6](#_ENREF_6)]

(ii) ’KEGG BLADDER CANCER’. In the clinical applications, a case has been reported sigmoid colon cancer could arise in a diverticulum when the urinary bladder involved [[7](#_ENREF_7)]. And for colorectal cancers that adhere to the urinary bladder, the partial or total cystectomy has been applied to achieve good local control [[8](#_ENREF_8)].

(iii) ’KEGG GLYCINE SERINE AND THREONINE METABOLISM’. As a key factor of hallmarkers of cancer, serine and glycine are biosynthetically associated (serine/glycine biosynthetic pathway) and relevant to the proliferation of cancer cell [[9](#_ENREF_9)], the hyperactivation of which would drive oncogenesis [[10](#_ENREF_10)].

**Table A2 The AUC of different rank lists for pathway genes (Colorectal cancer)**

| **PPI** | **STRING-based** | |
| --- | --- | --- |
| **Data** | **GSE25070** | **GSE8671** |
| All genes | 0.47011 | 0.42305 |
| DEGs | 0. 46597 | 0.46693 |
| twRWR | 0.83369 | 0.81326 |

**Table A3 The AUC of different rank lists for disease-associated genes (Colorectal cancer)**

| **PPI** | **STRING-based** | |
| --- | --- | --- |
| **Data** | **GSE25070** | **GSE8671** |
| All genes | 0.43769 | 0.41384 |
| DEGs | 0.45765 | 0.43782 |
| twRWR | 0.73745 | 0.68902 |

**A1.3 Dysregulated pathway-crosstalk identified to reveal the interactive map and module among pathways**

Similar to the analysis on Diabetes datasets, We first evaluated the pathway genes possibly selected by RWR，which uses the identified DEGs & DEVGs in a pathway as seeds. In the high-scored genes by RWR, there is a significant amount of pathway genes (seeing Table A2 and A3). Then by two-way RWR, we can find the interactive genes from two pathways, and select any pathway-pair as a crosstalk significantly (Table A5). All the crosstalks connect the known pathways as a map.

In the map of pathways, the modules of interactive pathways can be detected. Here, one biggest module can be seen, which is full of the pathways enriched with DEGs and DEVGs. By contrast, the pathways full of DEGs distribute in the map of pathways (Figure A2). Obviously, the pathways in this module are mostly related to metabolism, which supports the opportunity of using metabolites as diagnostic signatures or therapy targets for colorectal cancer [[11](#_ENREF_11), [12](#_ENREF_12)].

**
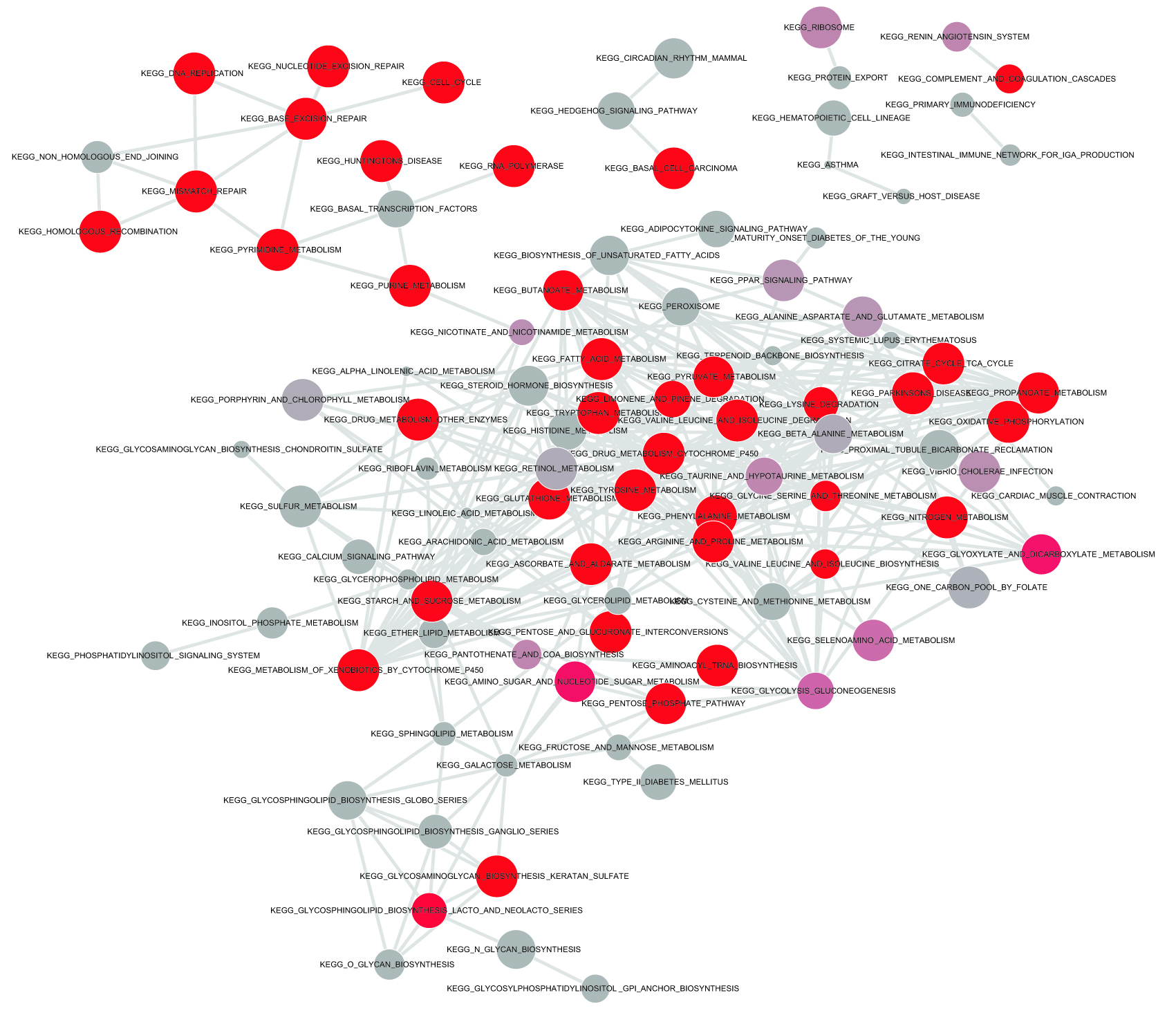
**

**Figure A2 The main topological structure of the map of pathways reconstrcucted on colorectal cancer datasets.**

**References:**

1. Hinoue T, Weisenberger DJ, Lange CP, Shen H, Byun HM, Van Den Berg D, Malik S, Pan F, Noushmehr H, van Dijk CM *et al*: **Genome-scale analysis of aberrant DNA methylation in colorectal cancer**. *Genome Res* 2012, **22**(2):271-282.

2. Sabates-Bellver J, Van der Flier LG, de Palo M, Cattaneo E, Maake C, Rehrauer H, Laczko E, Kurowski MA, Bujnicki JM, Menigatti M *et al*: **Transcriptome profile of human colorectal adenomas**. *Mol Cancer Res* 2007, **5**(12):1263-1275.

3. McIlwain CC, Townsend DM, Tew KD: **Glutathione S-transferase polymorphisms: cancer incidence and therapy**. *Oncogene* 2006, **25**(11):1639-1648.

4. Grubben MJ, Nagengast FM, Katan MB, Peters WH: **The glutathione biotransformation system and colorectal cancer risk in humans**. *Scand J Gastroenterol Suppl* 2001(234):68-76.

5. Liao C, Cao Y, Wu L, Huang J, Gao F: **An updating meta-analysis of the glutathione S-transferase T1 polymorphisms and colorectal cancer risk: a HuGE review**. *Int J Colorectal Dis* 2010, **25**(1):25-37.

6. Cotton SC, Sharp L, Little J, Brockton N: **Glutathione S-transferase polymorphisms and colorectal cancer: a HuGE review**. *Am J Epidemiol* 2000, **151**(1):7-32.

7. Yagi Y, Shoji Y, Sasaki S, Yoshikawa A, Tsukioka Y, Fukushima W, Hirosawa H, Izumi R, Saito K: **Sigmoid colon cancer arising in a diverticulum of the colon with involvement of the urinary bladder: a case report and review of the literature**. *BMC Gastroenterol* 2014, **14**:90.

8. Winter DC, Walsh R, Lee G, Kiely D, O'Riordain MG, O'Sullivan GC: **Local involvement of the urinary bladder in primary colorectal cancer: outcome with en bloc resection**. *Ann Surg Oncol* 2007, **14**(2):441-446.

9. Amelio I, Cutruzzola F, Antonov A, Agostini M, Melino G: **Serine and glycine metabolism in cancer**. *Trends Biochem Sci* 2014, **39**(4):191-198.

10. Locasale JW: **Serine, glycine and one-carbon units: cancer metabolism in full circle**. *Nat Rev Cancer* 2013, **13**(8):572-583.

11. Jacobs RJ, Voorneveld PW, Kodach LL, Hardwick JC: **Cholesterol metabolism and colorectal cancers**. *Curr Opin Pharmacol* 2012, **12**(6):690-695.

12. Leake I: **Colorectal cancer: Metabolic signature of CRC revealed by spectroscopic profiling**. *Nat Rev Gastroenterol Hepatol* 2013, **10**(9):503.
